# Metabolic stress-induced long ncRNA transcription governs the formation of meiotic DNA breaks in the fission yeast *fbp1 *gene

**DOI:** 10.1371/journal.pone.0294191

**Published:** 2024-01-22

**Authors:** Yusuke Tsuruta, Satoshi Senmatsu, Hana Oe, Charles S. Hoffman, Kouji Hirota

**Affiliations:** 1 Department of Chemistry, Graduate School of Science, Tokyo Metropolitan University, Hachioji-shi, Tokyo, Japan; 2 Biology Department, Boston College, Chestnut Hill, MA, United States of America; George Washington University, UNITED STATES

## Abstract

Meiotic recombination is a pivotal process that ensures faithful chromosome segregation and contributes to the generation of genetic diversity in offspring, which is initiated by the formation of double-strand breaks (DSBs). The distribution of meiotic DSBs is not uniform and is clustered at hotspots, which can be affected by environmental conditions. Here, we show that non-coding RNA (ncRNA) transcription creates meiotic DSBs through local chromatin remodeling in the fission yeast *fbp1* gene. The *fbp1* gene is activated upon glucose starvation stress, in which a cascade of ncRNA-transcription in the *fbp1* upstream region converts the chromatin configuration into an open structure, leading to the subsequent binding of transcription factors. We examined the distribution of meiotic DSBs around the *fbp1* upstream region in the presence and absence of glucose and observed several new DSBs after chromatin conversion under glucose starvation conditions. Moreover, these DSBs disappeared when *cis*-elements required for ncRNA transcription were mutated. These results indicate that ncRNA transcription creates meiotic DSBs in response to stress conditions in the *fbp1* upstream region. This study addressed part of a long-standing unresolved mechanism underlying meiotic recombination plasticity in response to environmental fluctuation.

## Introduction

Meiotic recombination between homologous chromosomes is essential for the faithful progression of meiosis. These recombination events generate crossovers that facilitate close contact to form bivalent chromosomes, which are pivotal for proper reductional chromosome segregation during meiosis I [1]. Moreover, meiotic recombination contributes to the augmentation of genetic diversity in gametes [2, 3], and the resultant diversity of offspring is thought to be important for the survival of a species in response to environmental fluctuations and to play an important role in evolution [4–6]. Whilst it has long been known that the distribution of recombination frequencies is influenced by environmental conditions, such as the temperature during gametogenesis [7–10], the mechanism underlying such changes in meiotic recombination frequencies has not been elucidated.

Meiotic recombination is initiated by the formation of double-strand breaks (DSBs) catalyzed by the conserved topoisomerase-like Spo11 protein (Rec12 in the fission yeast *Schizosaccharomyces pombe*) [11, 12]. The distribution of meiotic DSBs on the chromosome is not equivalent but clustered at hotspots. Mapping analyses of meiotic DSB sites in the budding yeast *Saccharomyces cerevisiae* [13] and in *S*. *pombe* [14] have revealed that meiotic DSB hotspots correlate with multiple factors, such as chromosome structure, chromatin configuration, histone modifications, and specific DNA sequences [12, 15–23]. Notably, some hotspots strongly correlate with the transcription start sites of mRNA and non-coding RNA (ncRNA), which generally have an open chromatin state, binding sites for transcription factors, and modified histones [22–28], suggesting a possible impact of transcription on the distribution of meiotic recombination.

The strict control of transcription is pivotal for all organisms to adapt to environmental changes [29]. The response of proper gene expression at the appropriate time is accomplished by the regulation of the binding of transcription factors to their cognate *cis*-acting DNA sequences within the promoter region of target genes [30–32]. Such interactions on DNA are under the regulation of chromatin structure; an array of nucleosomes composed of histone octamers, and genomic DNA [33]. Generally, chromatin structure plays a critical role in determining the transcription status, as the access of *trans*-acting factors to their target DNA is restricted by the tightly positioned chromatin [33, 34], and thus open (nucleosome-free) and closed (nucleosome-condensed) chromatin states are usually associated with transcriptional activation and gene silencing, respectively. In addition to this control, chromatin structure also plays a critical role in determining the sites of meiotic DSB formation, as the access of Spo11 to the target DNA is also restricted by tightly positioned nucleosomes [11, 35–40]. This view supports the hypothesis that transcriptional responses to various stresses during meiosis can affect the location of meiotic DSBs.

**Fig 1:**
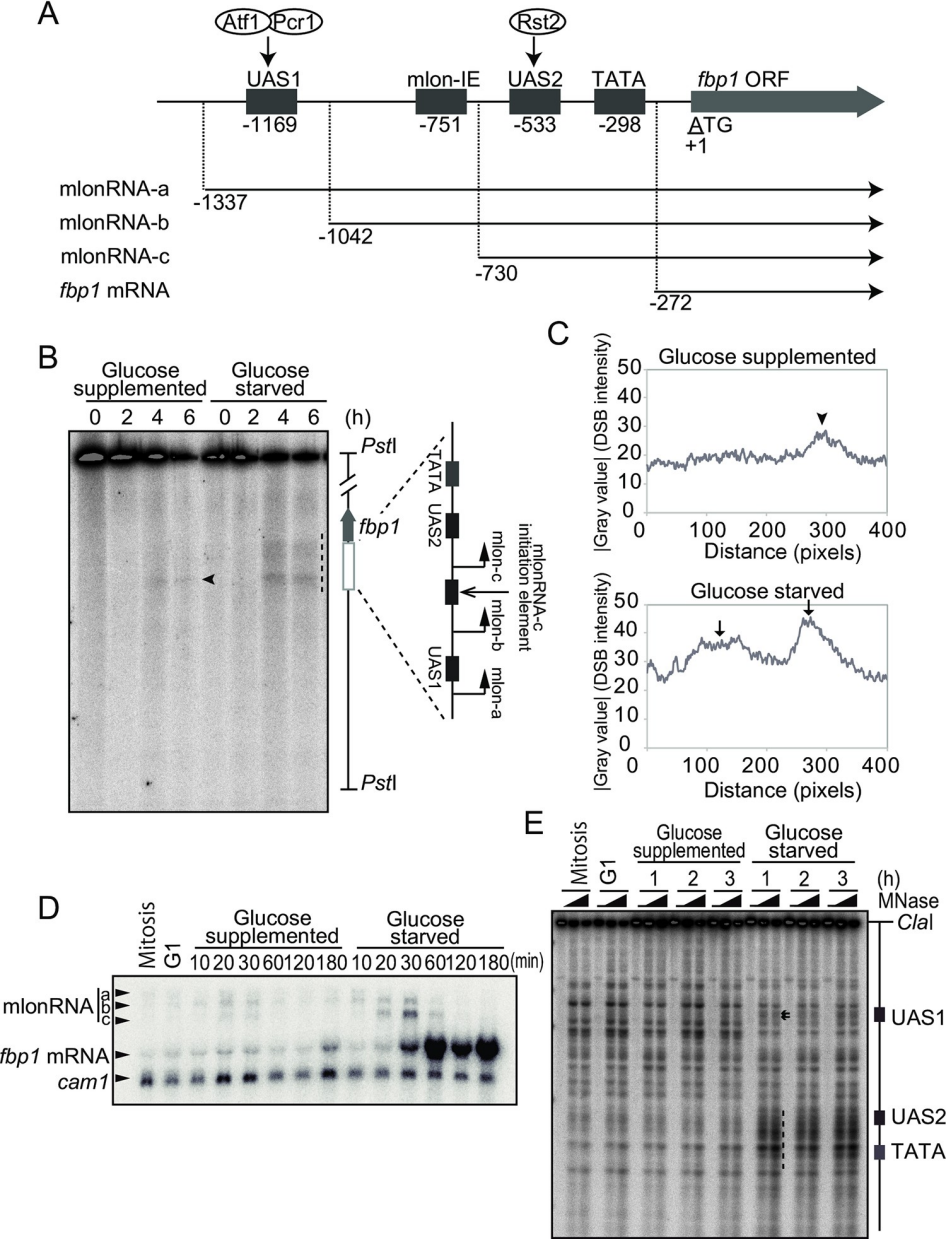
Formation of meiotic double-strand break (DSB) sites in the *fbp1* upstream region in response to glucose starvation stress. (A) Schematic representation of the *fbp1* upstream region containing upstream activating sequence 1 and 2 (UAS1 and UAS2), metabolic stress-induced long ncRNAs (mlonRNA)-c initiation element (mlon-IE), and TATA-box. UAS1 and 2 are the binding sites for Atf1-Pcr1 and Rst2, respectively. The numbers indicate the transcription start site of the *fbp1* transcripts and the distances of UAS1, UAS2, and TATA box from the first ATG of the *fbp1* open reading frame (ORF). (B) Haploid *pat1-114 rad50S* cells (SPH851) were cultured to induce meiosis in glucose-supplemented or glucose-starved conditions, and DNA was prepared as described in the Materials and Methods. Meiotic DSBs were detected by Southern blotting. The arrowhead indicates a faint DSB introduced in glucose-supplemented cells, while the dotted line indicates DSBs around UAS1 and UAS2 in glucose-starved cells. (C) Distribution of meiotic DSBs in glucose-supplemented or glucose-starved conditions. DSB band intensities in the *fbp1* upstream region six hours after the onset of meiosis (dotted line in B) were quantified using Image J (https://imagej.nih.gov/ij/, Plot Profile command). (D) Representative northern blot showing *fbp1* expression during the induction of meiosis with or without glucose. The lanes, Mitosis and G1 represent samples from asynchronous cell culture in YE medium and G1-arrested cells cultured in MM −N medium, respectively. The *cam1* transcript was used as an internal loading control. (E) Representative image of the chromatin analysis showing stepwise chromatin remodeling during glucose starvation. Haploid *pat1-114 rad50S* cells (SPH851) were induced for meiosis in the indicated condition, and cells were harvested at the indicated times.

In the fission yeast *S*. *pombe*, transcription of the *fbp1* gene, which encodes the gluconeogenic enzyme fructose-1,6-bisphosphatase, is massively activated in response to glucose starvation [41]. Prior *fbp1* gene activation, a series of ncRNAs referred to as metabolic stress-induced long ncRNAs (mlonRNAs; mlonRNA-a, mlonRNA-b, and mlonRNA-c) are transcribed upstream of the *fbp1* promoter [42] (Fig 1A). These transcriptional initiations induce chromatin remodeling [42–44], which in turn facilitates the binding of critical transcription factors, the bZIP heterodimer Atf1/Pcr1 [36, 45] and the zinc finger Rst2 [46] to upstream activating sequences 1 and 2 (UAS1 and UAS2), respectively [42, 47]. This leads to the transcription of *fbp1* mRNA in response to glucose starvation stress (Fig 1A). Thus, glucose starvation stress triggers a cascade of ncRNA transcription and induces an open chromatin configuration in the *fbp1* upstream region. It is further hypothesized that glucose starvation stress also affects meiotic DSB distribution in the *fbp1* upstream region *via* ncRNA transcription, since an open chromatin state induced by the ncRNA transcription would facilitate DSB formation. In this study, we examined this hypothesis and revealed mechanisms by which meiotic DSB formation is governed by ncRNA expression-mediated chromatin remodeling in response to environmental and metabolic stresses.

## Materials and methods

### Fission yeast strains, cell culture, and genetic methods

#### Fission yeast strains

S1 Table lists the fission yeast strains used in this study. Original strains carrying *UAS1-mut* or *UAS2-mut* genotype were generated previously [47]. Original cells lacking mlon-IE [44] or TATA-box [45] of *fbp1* gene were used.

#### Cell culture

YE medium (0.5% yeast extract and 2% glucose) and MM −N medium (0.3% KH phthalate, 0.22% Na_2_HPO_4_, 1% glucose, 2% salt stock [5.53% Mg_2_Cl_2_∙6H_2_O, 0.0735% CaCl_2_∙2H_2_O, 5.0% KCl, 0.2% Na_2_SO4], 0.1% 3-vitamin stock [0.1% Pantothenate Ca, 1% nicotinic acid, 1% inositol], 0.1% biotin stock [0.001% biotin], and 0.1% trace element [0.05% H3BO3, 0.53% MnSO_4_∙7H_2_O, 0.2% FeCl_3_∙6H_2_O, 1% (NH_4_)_6_Mo_7_O_24_∙4H_2_O, 0.1% KI, 0.04% CuSO_4_∙5H_2_O]) supplemented with required amino acids were used. MM+0.1N+Glu medium is an MM −N medium containing 1% YE and 0.1% NH_4_Cl, and MM+0.1N-Glu medium is an MM −N medium without 1% glucose containing 1% YE, 0.1% NH_4_Cl, 0.1% glucose and 3% glycerol. To detect meiotic DSBs, synchronous meiosis using a *pat1-114* mutation was employed together with a *rad50S* mutation to efficiently detect DSBs [37, 48]. Haploid *pat1-114 rad50S* cells were precultured in YE medium at 25°C, washed twice with distilled water, transferred to MM −N medium to arrest the cell cycle at the G1 phase, and cultured further at 25°C for 16 h. These arrested cells were collected by centrifugation, re-suspended in MM+0.1N+Glu medium (glucose-rich condition) or MM+0.1N-Glu medium (glucose starvation condition), and cultured at 34°C to induce meiosis.

#### Genetic methods

Plasmid transformation was performed using the lithium acetate method as previously described [49]. Briefly, 5 × 10^7^ cells from exponentially growing cultures were collected by centrifugation at 2330 g. Cells were washed with distilled water and 0.1 M Li-acetate in Tris-EDTA (TE; pH 7.5). Then, cells were resuspended in 40 μL of 0.1 M Li-acetate in TE (pH 7.5) and 5 μg of DNA construct and 300 μL of 40% PEG 4000 in 0.1 M Li-acetate in TE (pH 7.5) was added. The sample was agitated at 30°C for 30 min, followed by the addition of 40 μL of DMSO and incubation at 42°C for 15 min. For *pat1-114* cells, the cells were agitated at 25°C for 30 min and incubated at 25°C for 15 min. After centrifugation (2330 g), the cell pellet was resuspended in distilled water and plated on an appropriate selective medium. Mating and sporulation were performed on sporulation agar medium (0.1% KH_2_PO_4_, % glucose, 0.1% 3-vitamin stock, 0.1% viotin stock, and 3% agar) [50], followed by random spore analysis.

### Northern blotting

Northern blotting was performed as previously described, with slight modifications [35]. To prepare total RNA from *S*. *pombe* cells, 2 × 10^7^ cells were suspended in 0.3 mL of RNA extraction buffer (0.5 M NaCl, 0.2 M Tris-HCl [pH 7.5], 0.01 M ethylenediaminetetraacetic acid (EDTA), 1% SDS) and disrupted with 0.5 g glass beads and 0.3 mL of phenol-CHCl_3_ using Multi-Beads Shocker (Yasuikikai, Osaka Japan) at 2250 rpm for 30 s three times. After centrifugation of disrupted materials, the supernatants were extracted using phenol-CHCl_3_. *S*. *pombe* total RNA was isolated from 0.2 mL of the supernatant by ethanol precipitation and dissolved in 25 μL TE buffer (0.01 M Tris-HCl [pH 8.0], 0.001 M EDTA). Five micrograms of total RNA were denatured in a buffer (0.02 M MOPS [pH 7.0], 0.005 M sodium acetate, 0.001 M EDTA, 5.7% formaldehyde, 50% formamide) at 60°C for 5 min. The denatured sample was separated on 1.5% agarose gels containing formaldehyde by electrophoresis in a buffer (0.02 M MOPS [pH 7.0], 0.005 M sodium acetate, 1 m M EDTA) at 100 V for 2 h and transferred to a membrane (Biodyne B, PALL, NY). To detect *fbp1*, *cam1*, *rec6*, *rec8*, *and rec12* transcripts, a fragment of the *fbp1*, *cam1*, *rec6*, *rec8*, *and rec12* genes was amplified by PCR using the primers 5’-CGCCGATACAATCAGAAGC-3’ / 5’-CGATGAGTTTGCAGCATCC-3’ 5’-CTACCCGTAACCTTACAG-3’ / 5’-TGGAAGAAATGACACGAG-3’, 5’-AATGCGATGTCAAATGC-3’ / 5’-TCAGTCTGCAGCCGAA-3’, 5’-CCCAAAGCAGTTACTAC-3’ / 5’-CGAACATGTGAATCCTTG-3’, and 5’-CCAGATTTGATGACGAG-3’ / 5’-TACTGAGTGCTTTCCAC-3’, respectively. The resultant PCR fragments were used as a template for random-primer labeling (GE Healthcare) with ^32^P α-dCTP (PerkinElmer, MA) [51]. Hybridization was performed in a buffer (1% BSA, 7% SDS, 0.5 M Na_2_HPO_4_ [pH 7.4], 1 mM EDTA) at 62°C for 12 h and was extensively washed with wash buffer (1% SDS, 1 mM EDTA, 0.04 M Na_2_HPO_4_ [pH 7.4]). Signals were detected using a phosphor imager (FLA7000, Fuji Film, Tokyo).

### ChIP analysis

ChIP analyses were performed according to the method described by Senmatsu *et al*. [44] with slight modifications. Fifty milliliters of the culture were incubated with 1.4 mL of 37% formaldehyde solution for 20 min at room temperature, after which 2.5 mL of 2.5 M glycine was added. After centrifugation, collected cells were washed twice with ice-cold TBS buffer (150 mM NaCl and 20 mM Tris HCl [pH 7.5]). The cells were mixed with 400 μL of lysis 140 buffer (0.1% Na-deoxycholate, 1 mM EDTA, 50 mM HEPES-KOH [pH 7.5], 140 mM NaCl, and 1% Triton X100) supplemented with protease inhibitor, complete (Roche), and 0.6 mL of zirconia beads were added. After disruption of the cells using a Multi-Beads Shocker (Yasuikikai, Osaka), the suspension was sonicated six times for 30 s each on ice to shear chromosomal DNA into approximately 300–500 bp fragments, and centrifuged at 4°C. The supernatant was collected as a whole-cell extract. One microliter of Anti-Histone H3 antibody (Abcam, Cambridge, UK) or 1 μL of Anti-Atf1 antibody (Abcam, Cambridge, UK) and 10 μL of Dynabeads Protein A (Invitrogen, CA, USA) were mixed at 4°C to conjugate the antibody and beads and then was washed twice with PBS (138 mM NaCl, 2.7 mM KCl, 10 mM Na_2_HPO_4_, and 1.8 mM KH_2_PO_4_) containing 0.1% BSA. Finally, 180 μL whole-cell extract was mixed with pretreated beads and allowed to immunoprecipitate at 4°C for 16 h. The precipitates were washed twice with lysis 140 buffer, once with lysis 500 buffer (0.1% Na-deoxycholate, 1 mM EDTA, 50 mM HEPES-KOH [pH 7.5], 500 mM NaCl, and 1% Triton X100), and once with LiCl buffer (0.5% Na-deoxycholate, 1 mM EDTA, 250 mM LiCl, 0.5% NP-40, and 10 mM Tris-HCl [pH 8.0]) followed by washing once with TE (10 mM Tris-HCl [pH 8.0] and 1 mM EDTA). The well-washed precipitates were mixed with 40 μL of elution buffer (10 mM EDTA, 1% SDS, and 50 mM Tris-HCl [pH 8.0]) and allowed to elute the immunoprecipitated protein-DNA complexes at 65°C for 15 min (immunoprecipitation [IP] sample). To elute the IP sample completely, 100 μL of elution buffer and 150 μL of TE containing 0.67% SDS in the remaining beads were added and then incubated at 65°C for 15 min. Three microliters of whole cell extract were mixed with 97 μL of lysis 140 buffer and 400 μL of TE buffer containing 1% SDS (input sample). IP and Input samples were added to 84 μg proteinase K (Merck, Darmstadt, Germany) and incubated at 37°C for 16 h. After incubation, the temperature was increased to 65°C, and the sample was further incubated for 6 h. After incubation, DNA was extracted using phenol/chloroform from each sample and quantified by quantitative PCR using Thermal Cycler Dice Real Time (Takara Bio, Shiga, Japan) and THUNDERBIRD® SYBR qPCR Mix (TOYOBO, Osaka, Japan). The following primer sets, 5’-GGGATGAAAACAATCAACCTC -3’ / 5’-GGAATGCAGCAACGAAAATC-3’, 5’-GGGTGGAATGAGTCCGC-3’ / 5’-GTTCCGCGAATCATAAGCC-3’, and 5’-GCACAGTCGTTGTACAAATTCGTATTCCC-3’ / 5’-ACGATTCTAAACGCCTCTTGTTACGATCC-3’ were used for quantitative PCR analysis for UAS1, UAS2, and *prp3*, respectively.

### Micrococcal nuclease digestion assay

Chromatin structure was analyzed by indirect end-labeling according to a previously described method [43, 52], with modifications as follows. 5 × 10^8^ cells from 100 mL of the culture were harvested. Cells were incubated in 0.25 mL preincubation solution (20 mM Tris-HCl at pH 8.0, 0.7 M 2-mercaptoethanol, 3 mM EDTA) at 30°C for 10 min and washed once in 1 mL of ice-cold 1 M sorbitol with 10 mM EDTA. Cells were then centrifuged and resuspended in 1 mL of freshly prepared zymolyase solution (37.5 mM Tris-HCl at pH 7.5, 0.75 M sorbitol, 1.25% glucose, 0.1% (wt/vol) Zymolyase-100T, and 6.25 mM EDTA). The cells were incubated for 5 min at 30°C and the resulting spheroplasts were pelleted. The spheroplasts were then washed once in 1 mL ice-cold 1 M Sorbitol and resuspended well by pipetting 1 mL of freshly prepared lysis buffer (18% Ficoll-400, 10 mM KH_2_PO_4_, 10 mM K_2_HPO_4_ [pH 6.8], 1 mM MgCl_2_, 0.25 mM EGTA, 0.25 mM EDTA, and 1 mM phenylmethylsulfonyl fluoride (PMSF)). After centrifugation at 15,300 g for 30 min, the crude nuclear pellet was resuspended well in 1.5 mL of buffer A (10 mM Tris-HCl at pH 8.0, 150 mM NaCl, 5 mM KCl, 1 mM EDTA, and 1 mM PMSF). 0.5 ml aliquots of the crude chromatin suspension were digested with different concentrations of MNase (0, 20, and 50 U/ml) for 5 min at 37°C in the presence of 5 mM CaCl_2_. The reaction was terminated by adding 40 mM EDTA, 1% SDS, and 200 μg of proteinase K and incubated at 55°C for 16 h. The insoluble material was removed by centrifugation at 4°C. The supernatants were extracted with phenol/chloroform, digested with RNase A, and extracted once with phenol/chloroform. The extracted DNA was precipitated by 2-propanol, rinsed in 70% ethanol, and resuspended in 30 μL TE buffer. DNA samples were digested by ClaI for analysis of the *fbp1* region and separated by electrophoresis on 1.5% agarose gel in Tris base, acetic acid, and EDTA (TAE) buffer (0.48% Tris-base, 0.11% acetic acid, and 0.037% EDTA) at 60 V for 20 h, followed by Southern blotting using the same probe as in the detection of *fbp1* transcripts. The methods for hybridization and signal detection were the same as those in northern blot analysis.

### Detection of meiotic DSBs

DNA samples were prepared in agarose plugs from cells from a synchronous meiotic culture according to the method described by Hirota *et al*. [38] with the following modifications. Briefly, 50 mL of the culture was harvested and washed twice with 500 μL of CSE buffer (20 mM citrate-phosphate (pH 5.6), 1.2 M sorbitol, and 40 mM EDTA). After resuspension with 500 μL of CSE buffer containing 1.5 mg/mL Zymolyase 20T, cells were incubated at 37°C for 60 min, centrifuged, and resuspended in 500 μL of TSE buffer (10 mM Tris-Cl (pH 7.5), 0.9 M sorbitol, and 45 mM EDTA). An equal volume of low-melting-point agarose (1%) was added in TSE buffer, and the mixture was poured into agarose plug molds (Bio-Rad Laboratories, Hercules, CA, USA). After 5 min on ice, the agarose plugs were collected into lysis buffer (0.25 M EDTA, 50 mM Tris-Cl [pH 7.5], and 1% sodium dodecyl sulfate [SDS]) and incubated at 55°C for 1.5 h. Then, the lysis buffer was removed, and a second lysis buffer (1% lauryl sarcosine, 0.5 M EDTA [pH 9.5], and 1 mg/mL of proteinase K) was added. The mixture was incubated at 55°C for 48 h. Next, agarose plugs were soaked in 1 mL TE containing 0.5 mM PMSF at room temperature for 1 h and washed twice with TE at room temperature for 1 h. The agarose plugs were equilibrated using CutSmart buffer (New England Biolabs, Ipswich, MA, USA) for 16 h. After changing the CutSmart buffer, the agarose plugs were incubated at room temperature for 1 h. DNA in the agarose plugs was digested by PstI or AflII (New England Biolabs) to detect meiotic DSBs in *fbp1* or *ade6-M26*, respectively. The digested DNA fragments were separated by electrophoresis on 0.8% agarose gel in TAE buffer at 60 V for 20 h, followed by Southern blotting. To detect DSBs in the *fbp1* upstream region and *ade6-M26*, a fragment of the *fbp1* and *ade6* gene was amplified by PCR using the primers 5’-GTGAAACCCTGATATTCTGAG-3’ / 5’-GGAGCAACGTAGCTTGG-3’ and 5’-CCATTTGTGGGAACCAATATG-3’ / 5’-GCTCGTTTAAATGCTAAACC-3’, respectively. The methods for hybridization and signal detection were the same as those in northern blot analysis. To detect whole meiotic DSBs, the agarose plugs were washed with TE, and the chromosomal DNA and DSBs in the agarose plugs were separated by pulsed-field gel electrophoresis (PFGE) and stained with ethidium bromide. PFGE was carried out in 0.8% chromosomal grade agarose on a CHEF-DRIII system (Bio-Rad Laboratories, Hercules, CA, USA) and re-circulated at 14°C. Electrophoresis was performed for 48 h at 2 V/cm in 1 × TAE (40 mM Tris, 20 mM acetate, 1 mM EDTA) buffer, with a switch time of 30 min at an included angle of 100°.

### Antibodies

Anti-Histone H3 antibody (ab1791, Lot:1025144, Abcam, Cambridge, UK) and Anti-Atf1 antibody (ab18123, Lot:GR218223-3, Abcam, Cambridge, UK) were used.

### Statistics, reproducibility, and data representation

The sample mean and standard deviation values are shown on each bar graph. All statistical analyses were conducted using a one-sided Student’s *t*-test. A one-sided *p*-value < 0.05 was considered statistically significant, as two groups of the data were assessed for their significant difference in this study.

## Results

### Formation of meiotic DSB sites in the *fbp1* upstream region in response to glucose starvation stress

To investigate the possible roles of ncRNA expression in the determination of meiotic DSB sites, we compared the frequency and distribution of meiotic DSBs in the *fbp1* upstream region in the presence and absence of glucose. In the following experiments, genomic DNA was isolated from *pat1-114 rad50S* haploid cells, which are useful to analyze meiotic DSB since the *pat1-114* mutation enables synchronous meiosis by temperature shift [53, 54] and the *rad50S* mutation helps DSB detection by arresting DSB repair [48]. We induced meiosis in glucose-supplemented and glucose-starved conditions. The meiotic nuclear division was critically impaired in the glucose-starved condition, whereas expression of meiotic genes was preserved in glucose-starved meiosis (S1A and S1B Fig). In the presence of glucose, we found a faint DSB signal around UAS1 in the *fbp1* upstream region four and six hours after the induction of meiosis (Fig 1B and 1C, arrowhead). In contrast, at least two prominent meiotic DSB signals around UAS1 and UAS2 appeared in glucose-starved cells (Fig 1B and 1C, dotted line in B, and arrows in C). Despite these changes of the distribution pattern of DSBs in the *fbp1* upstream region in response to glucose starvation stress, the DSB distribution was preserved in the control DSB hotspot *ade6-M26*, a gene that does not respond to glucose starvation [55] (S1C Fig). Since the recombination in *ade6-M26* is not fully activated in synchronous meiosis in a *pat1-114* background [56] and these control data seem not to be particularly convincing, we analyzed total DSBs using pulsed-field gel electrophoresis. The total DSBs also seem to remain unchanged upon glucose starvation (S1D and S1E Fig). These results indicate that the formation of meiotic DSB sites is specifically induced in the *fbp1* upstream region in response to glucose starvation. To address the involvement of ncRNA transcription in the formation of meiotic DSBs, we examined the expression profile of mlonRNAs and mRNA under glucose starvation stress during meiosis. To synchronously induce meiosis, we cultured cells in a medium lacking a nitrogen source to arrest the cell cycle in the G1 phase and released them in meiotic conditions at time 0. As nitrogen starvation induces the MAPK pathway that activates Atf1-Pcr1[57, 58], this may be responsible for leaky expression of mlonRNAs and mRNA observed prior to glucose starvation during meiosis (Fig 1D). Upon entry into meiosis, each mlonRNA (a, b, and c) and mRNA of *fbp1* was induced in a stepwise manner during glucose starvation during meiosis (10–60 min of glucose starvation), with no such induction observed under glucose-supplemented conditions (Fig 1D). We also analyzed the chromatin structure in the *fbp1* upstream region by investigating the distribution of micrococcal nuclease (MNase) sensitive sites (Fig 1E). The distribution patterns of MNase-sensitive sites in mitotic, G1-arrested, and meiotic cells in glucose-supplemented conditions were comparable, whereas meiotic cells under glucose starvation conditions exhibited drastic changes in the distribution pattern. New MNase-sensitive bands appeared at UAS1 (Fig 1E, arrows) and intense MNase-sensitive bands appeared around the UAS2–TATA box region at 60–180 min after glucose starvation (Fig 1E, dotted line), which coincided with the robust *fbp1* mRNA transcription. (Fig 1D and 1E). Collectively, these results indicate that the transcription of *fbp1* mlonRNA/mRNA in response to glucose starvation stress is associated with chromatin conversion, and this transcription-associated chromatin change induces the subsequent meiotic DSB formation in the *fbp1* upstream region.

### The formation of meiotic DSBs in *fbp1* is mediated by mlonRNA initiations

**Fig 2:**
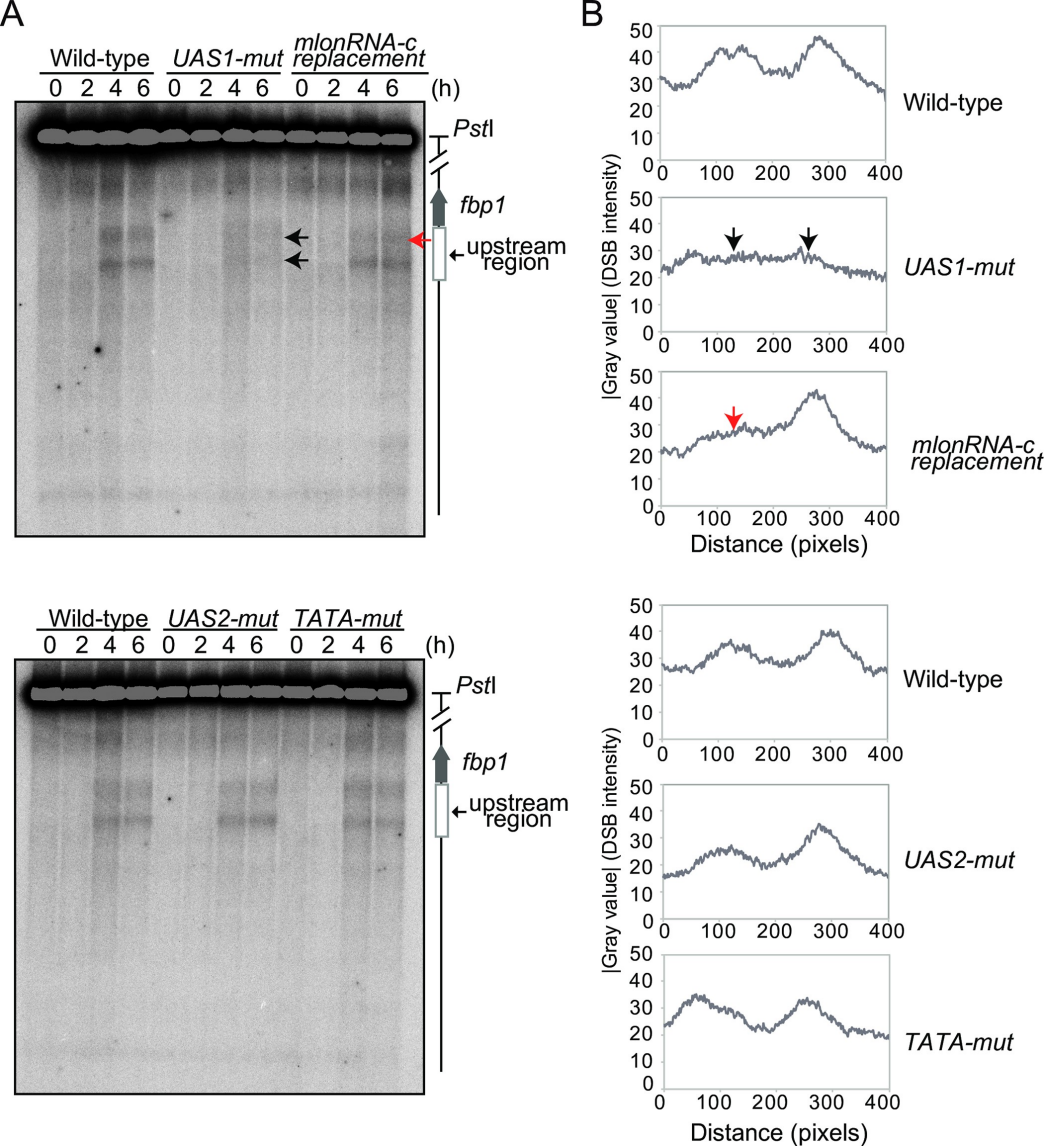
UAS1 and mlonIE elements are required for DBS formation in the *fbp1* upstream region upon glucose starvation. (A) Haploid *pat1-114 rad50S* cells possessing the indicated genotype (wild-type: SPH851, *UAS1-mut*: SPH832, *UAS2-mut*: SPH850, *TATA-mut*: SPH816, and *mlonRNAc-replacement*: SPH815) were cultured to induce meiosis in glucose starvation conditions, and DNA was prepared and meiotic DSBs were detected as in Fig 1B. The black and red arrows indicate the lost DSB sites in *UAS1-mut* and *mlonRNA-c-replacement* cells. (B) Distributions of meiotic DSBs induced at six hours after the onset of meiosis under glucose starvation in cells with the indicated genotype were quantified using Image J as in Fig 1C.

Next, we investigated whether mlonRNA or mRNA transcription is involved in the introduction of meiotic DSBs in the *fbp1* upstream region. To this end, we mutated several critical *cis*-elements required for transcription of these various RNAs. We analyzed mutant cells carrying a point mutation in UAS1 or UAS2 [47], the critical binding sites for transcription factors Atf1-Pcr1 [36, 45] and Rst2 [46], respectively. Both elements are required for *fbp1* mRNA expression, while UAS1, but not UAS2, is required for the induction of mlonRNA [42, 44, 52]. In addition to these two *cis*-elements, we analyzed the mlonRNA initiation element (mlon-IE), which is involved in mlonRNA-c transcription [35, 44]. Moreover, we analyzed the *fbp1* TATA box, which is required for *fbp1* mRNA expression but not for the induction of mlonRNA [45]. The cells carrying a mutation in UAS2 (*UAS2-mut*) and cells lacking the *fbp1* TATA box (*TATA-mut*) showed a very similar DSB distribution compared to wild-type cells under glucose starvation conditions (Fig 2A and 2B), indicating that the expression of *fbp1* mRNA is not important for the creation of DSB sites in the *fbp1* upstream region upon glucose starvation stress. In contrast, cells with a mutation in UAS1 (*UAS1-mut*) showed substantial changes in the distribution of DSBs around UAS 1 and UAS2 compared to wild-type cells (Fig 2A and 2B; black arrows). Moreover, mutant cells in which mlon-IE was replaced with a part of the *act1* sequence (*mlonRNA-c-replacement*) also showed a change in the distribution of DSBs around UAS2 compared to wild-type cells (Fig 2A and 2B; red arrows). These results indicate that the induction of mlonRNAs rather than *fbp1* mRNA is required for meiotic DSB formation in *fbp1* in response to glucose starvation stress.

### mlonRNA transcription facilitates chromatin remodeling in the *fbp1* upstream region

**Fig 3:**
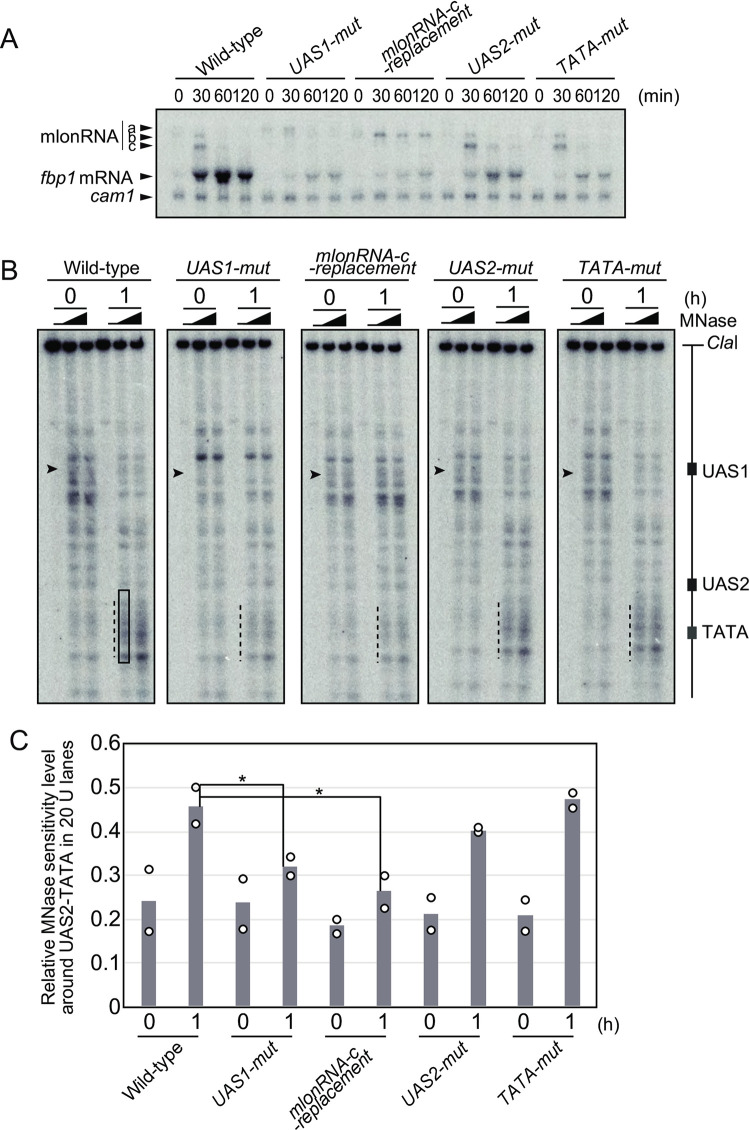
Important role of UAS1 and mlon-IE elements in mlonRNA transcription and chromatin remodeling in the *fbp1* upstream region. (A) Representative image of a northern blot showing *fbp1* expression during the induction of meiosis in the glucose starvation condition. Cells with the indicated genotype were assessed (wild-type: SPH851, *UAS1-mut*: SPH832, *UAS2-mut*: SPH850, *TATA-mut*: SPH816, and *mlonRNAc-replacement*: SPH815). Cells were cultured to induce meiosis for the indicated time, as described in Fig 1B. The *cam1* transcript was used as an internal control. (B) Representative images of the chromatin analyses showing chromatin configuration changes in the *fbp1* upstream region during meiosis under glucose starvation conditions. (C) Quantification of MNase-sensitive bands around UAS2-TATA. Band intensity was quantified using Image J. Levels of MNase sensitive bands in the UAS2-TATA region (black square in B) relative to that of whole signals in a lane is shown. *n* = 2 biologically independent experiments. Student’s *t*-test, *p < 0.05.

Both the *UAS1-mut* and *mlonRNA-c-replacement* mutants exhibited defects in the formation of meiotic DSBs in the *fbp1* upstream region in response to glucose starvation stress (Fig 2A), but these mutants showed distinctly different defects; *UAS1-mut* cells exhibited the changes of DSB-distributions around both UAS1 and UAS2, whereas *mlonRNA-c-replacement* cells showed a change of DSB-distribution only at UAS2. We next investigated the mechanisms underlying this difference by analyzing the *fbp1* transcription profile and chromatin structure (Fig 3A). Consistent with previous studies [44, 45, 47], inactivation of each *cis*-element diminished *fbp1* mRNA transcription (Fig 3A). More importantly, *UAS1-mut* and *mlonRNA-c-replacement* cells exhibited distinct defects in mlonRNA induction; *UAS1-mut* cells showed little expression of mlonRNA-b, with prolonged transcription of mlonRNA-a (0–30 min) and impaired induction of mlonRNA-c, whereas *mlonRNA-c-replacement* cells showed prolonged mlonRNA-b expression (30–120 min) and a defect in the expression of mlonRNA-c (Fig 3A). We next examined whether the inactivation of these *cis*-elements influences chromatin remodeling in *fbp1*. As expected, *UAS1-mut* and *mlonRNA-c-replacement* cells showing defects in mlonRNA-c expression exhibited impaired chromatin remodeling around the UAS2–TATA box region, whereas *UAS2-mut* and *TATA-mut* cells were able to alter the chromatin structure around the UAS2–TATA box region (Fig 3B and 3C, indicated by dotted lines). Notably, *UAS1-mut* cells but not *mlonRNA-c-replacement* cells showed defects in inducing MNase-sensitive sites at UAS1 (Fig 3B, arrowheads). These results indicate that UAS1 is required for chromatin conversion events at UAS1 and UAS2–TATA, whereas mlon-IE is required for chromatin remodeling at UAS2–TATA.

**Fig 4:**
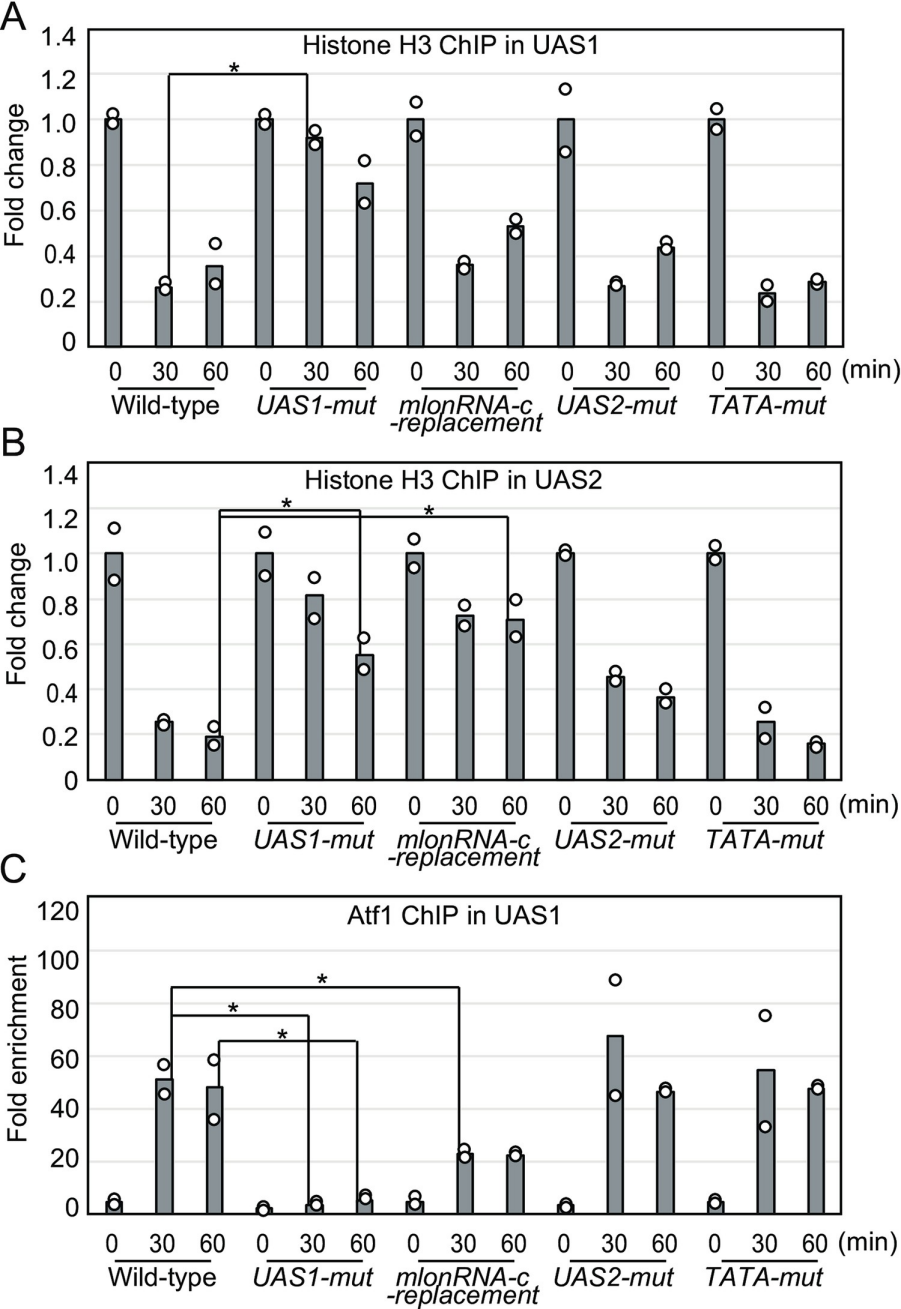
UAS1 and mlon-IE elements are required for Histone H3 eviction from the *fbp1* upstream region and Atf1 binding to UAS1. (A, B) Chromatin immunoprecipitation (ChIP) analysis to examine histone H3 binding at UAS1 (A) and UAS2 (B) in the indicated cells (wild-type: SPH851, *UAS1-mut*: SPH832, *UAS2-mut*: SPH850, *TATA-mut*: SPH816, and *mlonRNAc-replacement*: SPH815). Cells were cultured to induce meiosis for the indicated time, as described in Fig 1B. ChIP signals were quantified by qPCR. ChIP signals for the *prp3* ORF were used for normalization. Fold changes are shown for each genotype. *n* = 2 biologically independent experiments. Student’s *t*-test, *p < 0.05. (C) ChIP analysis to examine Atf1 binding at UAS1. Cells were cultured to induce meiosis for the indicated time, as described in Fig 1B. Fold enrichment was calculated by normalizing with ChIP signals for the *prp3* ORF. *n* = 2 biologically independent experiments. Student’s *t*-test, *p < 0.05.

To confirm these results, we performed histone H3 chromatin immunoprecipitation (ChIP) analyses of the UAS1 and UAS2 regions (Fig 4A and 4B). Consistent with the results of the chromatin analyses using MNase, wild-type, *UAS2-mut*, and *TATA-mut* cells showed a reduction in histone occupancy in both UAS1 and UAS2 regions after glucose starvation stress, indicating that efficient chromatin remodeling occurs around these regions in these cells (Fig 4A and 4B). In sharp contrast, both *UAS1-mut* and *mlonRNA-c-replacement* cells exhibited significantly reduced kinetics of histone eviction at UAS2 compared to wild-type cells (Fig 4B). Moreover, *UAS1-mut* cells but not *mlonRNA-c-replacement* cells, exhibited high histone occupancy in the UAS1 region after glucose starvation stress (Fig 4A). These results indicate that transcription of mlonRNAs induces chromatin remodeling around each initiation site of mlonRNAs. Taken together, metabolic stress-induced mlonRNA transcription appears to induce the formation of meiotic DSBs through chromatin remodeling.

We further examined whether alteration of these *fbp1 cis-*elements influences the binding of Atf1 as all meiotic DSBs in the *fbp1* upstream region required the Atf1 binding sequence, UAS1 (Fig 2). As expected, *UAS1-mut* cells exhibited impaired Atf1 binding (Fig 4C). In contrast, *UAS2-mut* and *TATA box-mut* cells exhibited Atf1 binding at wild-type levels (Fig 4C). *mlonRNA-c-replacement* cells exhibited a partial reduction in Atf1 binding at UAS1 compared to wild-type cells (Fig 4C). These results are consistent with the antagonistic function of mlonRNA-c transcripts against the repressive function of Tup1-family corepressors, Tup11/12, with regard to Atf1 binding [59]. Thus, it is also possible that not only chromatin remodeling mediated by mlonRNA transcription but also mlonRNA molecules themselves contribute to the formation of meiotic DSBs upon metabolic stress by stabilizing Atf1 binding.

### Pivotal roles of the transcription factor Atf1-Pcr1 in the formation of meiotic DSBs in the *fbp1* upstream region

**Fig 5:**
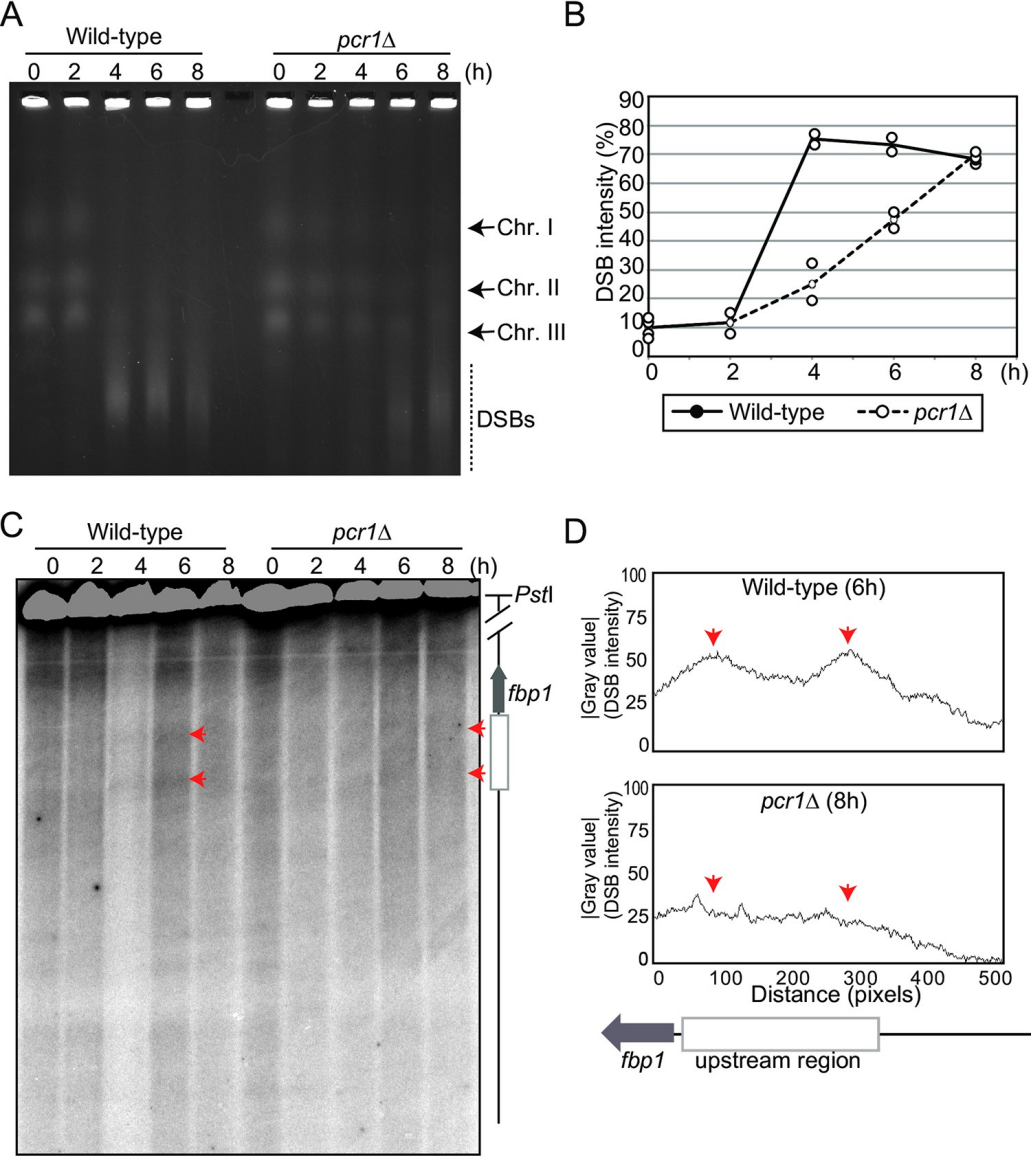
Atf1-Pcr1 transcription factor is required for the formation of meiotic DSBs under glucose starvation conditions. (A) Whole meiotic DSBs induced in the indicated cells were analyzed by pulsed-field gel electrophoresis (wild-type: SPH851, *pcr1Δ*: SPH887). The dotted line indicates DSB signal. (B) Intensities of DSB signals in the indicated cells were quantified using Image J (C) DSBs in the *fbp1* upstream region were analyzed as in Fig 1B. Red arrowheads represent the sites of DSBs induced around UAS1 and UAS2. (D) DSB intensity was quantified as in Fig 1C.

As described above, the Atf1-Pcr1 binding site (UAS1) is essential for mlonRNA transcription, chromatin remodeling, and the formation of meiotic DSBs in response to glucose starvation. This suggests that the transcription factor Atf1-Pcr1 may serve as the master regulator for the redistribution of meiotic DSBs under stress conditions. Thus, we examined the importance of the Atf1-Pcr1 transcription factor in meiotic DSB formation in the *fbp1* upstream region upon glucose starvation. We used *pcr1*Δ cells to test this hypothesis as Atf1 is required for cell cycle arrest at the G1 phase (an entry phase for sexual development) and cells deficient in Atf1 show poor meiotic progression compared to wild-type cells [38, 57]. *pcr1*Δ cells displayed a slower rate of total DSB formation than wild-type cells, with similar total DSB intensity for wild-type cells at six hours and *pcr1Δ* cells at eight hours (Fig 5A and 5B). We then compared meiotic DSB distribution in the *fbp1* upstream region at six hours for wild-type cells and eight hours for *pcr1Δ* cells (Fig 5C and 5D). Wild-type cells showed prominent DSBs around UAS1 and UAS2 in the *fbp1* upstream region, whereas *pcr1*Δ cells exhibited little DSB signal similar to that of *UAS1-mut* cells, indicating that meiotic DSB formation in *fbp1* upon glucose starvation stress is dependent on the Atf1-Pcr1 transcription factor. Taken together, we conclude that the Atf1-Pcr1 transcription factor serves as a critical mediator for the formation of meiotic DSBs upon metabolic stresses in the *fbp1* upstream region.

## Discussion

In this study, we demonstrate that meiotic DSBs are induced in the *fbp1* upstream region by glucose starvation stress (Fig 1). This DSB induction under environmental stress is mediated by chromatin remodeling induced by ncRNA transcription (Figs 2–4). We show a critical role for the transcription factor Atf1-Pcr1 in the formation of DSB sites under stress conditions.

**Fig 6:**
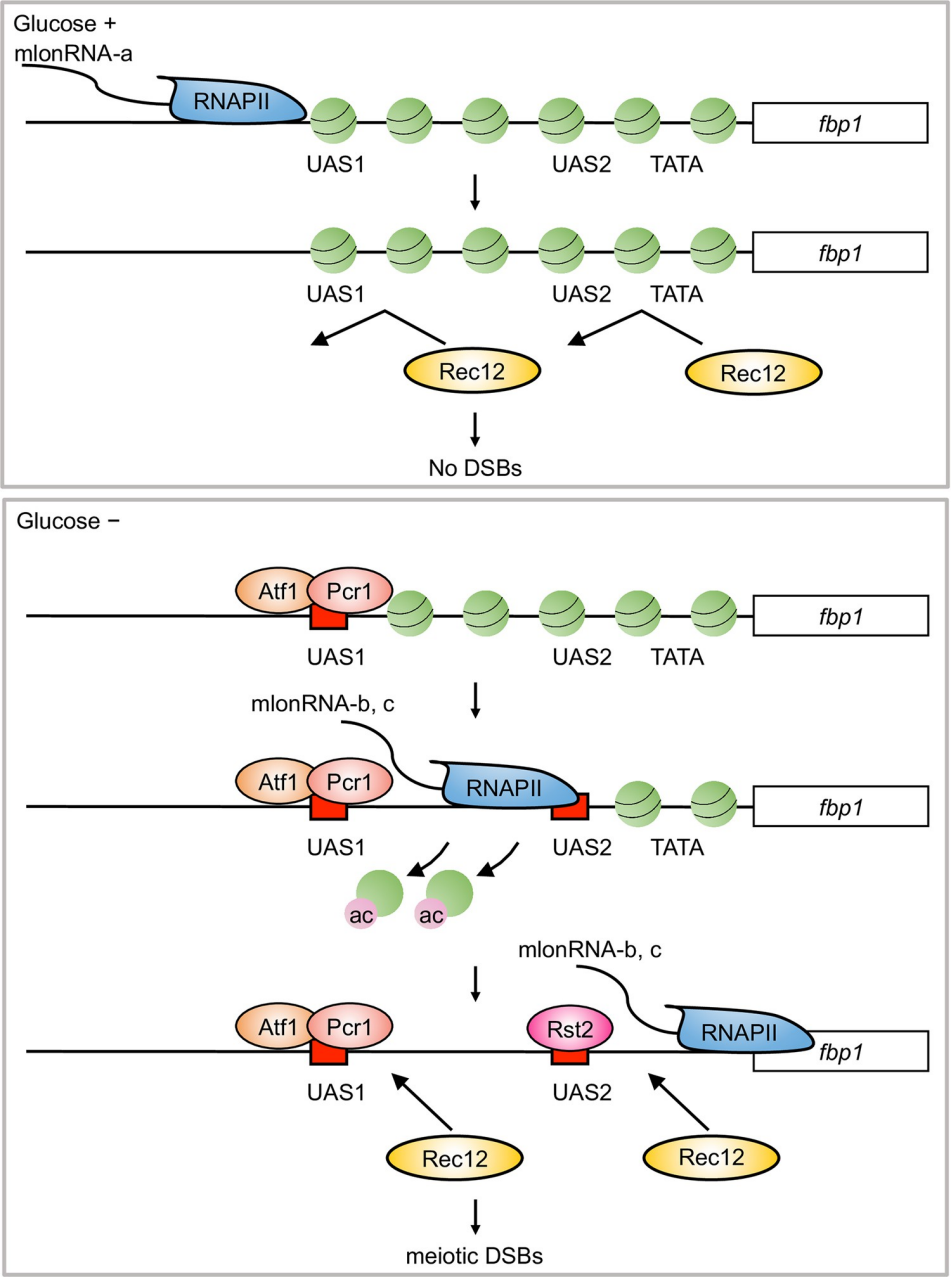
Model of mlonRNA transcriptional initiation mediated DSB formation. Schematic illustrations of the mechanism of DSB formation via mlonRNA expression under glucose-starvation conditions are shown in comparison to the lack of DSB formation under glucose-rich conditions.

Based on these findings, we propose the following model in the formation of DSBs in the *fbp1* upstream region under glucose starvation stress (Fig 6). When cells enter meiosis in the presence of glucose, the *fbp1* upstream region remains protected from access by transcription factors and Rec12 (Spo11 homolog in *S*. *pombe*) due to tightly positioned nucleosomes (Fig 6, Glucose +). Thus, Rec12-mediated DSB formation is restricted. In contrast, in the absence of glucose (Glucose −), stepwise transcription of ncRNAs (mlonRNA-a, b, and c) in the *fbp1* upstream region are initiated by the Atf1-Pcr1 transcription factor. The factors bound to mlon-IE and required for mlonRNA-c initiation have not been elucidated. Subsequently, the progression of RNA-Polymerase II transcription of mlonRNAs converts the chromatin configuration into an open chromatin state, which allows access of transcription factors and Rec12 to DNA, leading to the introduction of DSBs. These responses to stress conditions through ncRNA transcription may account for the plasticity of meiotic DSBs upon environmental fluctuations.

The formation of meiotic DSBs in *fbp1* upon glucose starvation is dependent upon the Atf1-Pcr1 transcription factor (Fig 4). The *ade6-M26* meiotic recombination hotspot is also an Atf1-Pcr1 transcription factor mediated-hotspot (reviewed in [11]), but not in a glucose-regulated manner. It remains unclear how cells select meiotic recombination hotspots among the numerous Atf1-Pcr1 binding sites. Previous statistical analyses have shown that only 19% of Atf1-Pcr1 binding motifs in the fission yeast genome are hotspots [14], suggesting that an Atf1-Pcr1 binding sequence is not sufficient to create a hotspot, and other factors are needed to create meiotic DSBs. It is possible that Atf1-Pcr1 binding sites without meiotic DSBs can be changed into hotspots by environmental stresses that mediate chromatin remodeling to promote Atf1-Pcr1 binding. Chromatin remodeling associated with ncRNA transcription might contribute to the formation of meiotic DSBs in cold regions carrying an Atf1-Pcr1 binding site. Consistent with this idea, insertion of the mlon-IE sequence into the *ade6-M26* gene facilitates chromatin remodeling and additional meiotic DSB formation downstream of the Atf1-Pcr1 binding site of *ade6-M26* [35]. Therefore, the collaboration between Atf1-Pcr1 and mlonRNA transcription may facilitate meiotic DSBs in response to environmental cues, thereby contributing to recombination plasticity in stressed conditions.

Most of the previous studies on meiotic DSB in *S*. *pombe* have used *pat1-114* strains, in which the Pat1 kinase can be inactivated by high-temperature to induce meiosis in a synchronous manner [54, 56, 60, 61]. The disadvantage of the use of synchronous meiosis in a *pat1-114* background is that meiotic DSB formation in some regions could be affected due to the absence of a second mating type gene [56, 61]. The recently improved protocol employs *Mat1-Pc* expression in *h*^*-*^ *pat1-114* cells harboring *Mat1-Mc* to increase the synchrony of meiosis [54], and the use of this method might be required for the accurate investigation of meiotic DSB. The use of *pat1-114* requires heat shock stress to induce synchronous meiosis. Thus, this strain is not suitable for the analysis of individual stress responses during meiosis. This problem could be resolved by another *pat1*-conditional inactivation strain, *pat1-ATP analog sensitive mutant* (*pat1-as*) [62, 63], as the *pat1-as* mutant allows for the induction of synchronous meiosis at physiological temperatures by adding the ATP analogs, 1-NM-PP1 or 3-BrB-PP1. Hence, further analyses using *pat1-as* mutants are necessary to clarify meiotic recombination plasticity in response to individual environmental conditions.

This study shows that stepwise mlonRNA transcription induces meiotic DSBs in the *fbp1* upstream region through chromatin remodeling, under glucose starvation conditions. Our previous studies have shown that histone H3 acetylation occurring around mlonRNA initiation sites is involved in chromatin remodeling [43], similar to what is observed in another Atf1-Pcr-mediated meiotic recombination hotspot, *ade6-M26* [24, 37, 40]. In mammals, the histone methyltransferase PRDM9 is pivotal for determining meiotic DSB sites [64, 65]. PRDM9 binds to specific DNA sequences, thereby inducing trimethylation of histone H3 at lysine 4 (H3K4me3) and lysine 36 (H3K36me3) in neighboring nucleosomes. Indeed, meiotic recombination hotspots in mammals, including humans and mice, strongly correlate with H3K4me3 levels [65]. Similarly, meiotic recombination hotspot activity in budding yeast is regulated by H3K4me3 induced by the Set1 H3K4-trimethyltransferase [15, 66]. In contrast to these organisms, no significant overlap between meiotic recombination hotspots and H3K4me3 has been detected in *S*. *pombe*, while histone H3-K9 acetylation (H3K9ac) is significantly enriched in the hotspots [21], suggesting a dominant and universal role of histone acetylation in the regulation of meiotic DSB distribution. In *S*. *pombe*, these modifications (H3K9ac and H3K4me3) might carry out different roles in the regulation of DSBs, as the loss of either H3K9ac or H3K4me3 has different effects on DSB formation at the site of induction [21]. Taken together, H3K9ac and H3K4me3 modifications may serve as a guide for meiotic DSB sites, but their relative usage may differ among organisms. These differences in the usage of epigenetic codes for the determination of meiotic DSBs might be due to differences in the expression and roles of ‘reader proteins’ that decode epigenetic signals in organisms.

Evolution is a continuous process that involves genetic variation and phenotypic selection [67]. At a molecular level, mutations in genes cause diversity in the functionality of genes, and it is believed that alleles possessing advantages that fit environmental conditions are selected from such diverse alleles [68]. Meiotic recombination produces genetic diversity in gametes via genome-shuffling between parental genomes [2, 3], and the resultant diversity in offspring might play an important role in evolution [4–6]. In the current study, we show that transcription events induced by environmental changes trigger meiotic DSB formation events. These events might be induced around gene promoter regions, since transcription factor-binding elements are usually located in such non-coding regions, and thus DNA sequences not only within the gene body but also in the promoter region should be efficiently diversified. This view is consistent with the recent study showing the pivotal role of mutations in non-coding regulatory DNA sequences such as promoter sequences in organismal phenotype and fitness [69]. Moreover, frequent recombination-mediated genome-shuffling between homologous DNAs at the actively-transcribed genes in response to various stresses might facilitate rapid molecular evolution in response to environmental stresses.

This study revealed a subset of mechanisms that can contribute to the plasticity of meiotic DSBs in response to environmental stresses. Further research on the plasticity of meiotic recombination will be important for understanding the evolutionary mechanisms that allow organisms to adapt to environmental changes.

## Supporting information

S1 Table Fission yeast strains used in this study. (PDF)

S1 Fig Normal meiotic DSB formation during meiosis under glucose starvation stress. (A) Meiotic progressions of *h*^*-*^ *pat1-114* cells (SPH731) in glucose-supplemented and glucose-starved conditions were monitored by the observation of nuclear divisions. (B) Representative images of the Northern blot showing the expression of meiotic genes (*rec6*, *rec8*, and *rec12*) after the onset of meiosis with or without glucose. The *cam1* transcript was used as an internal control. (C) Haploid *pat1-114 rad50S* cells (SPH851) were cultured to induce meiosis with or without glucose as in Fig 1B. Meiotic DSBs around the *ade6-M26* locus were detected by Southern blotting. The arrow indicates the position of *M26* mutation sites where the Atf1-Pcr1 transcription factor binds and induces DSBs. (D) Whole genome DNA including each intact chromosomal DNA and DSBs were separated with pulsed-field gel electrophoresis as in Fig 5. Dotted line indicates DSB signal. (E) Intensities of DSB signals in the indicated cells were quantified using Image J.
(PDF)

S2 Fig Uncropped images. Full size images of raw blot data were shown in S1 Raw images.
(PDF)

S1 Raw images Full size images of raw blot data used in this study. (PDF)
