# Supplementary material for: Metabolic stress-induced long ncRNA transcription governs the formation of meiotic DNA breaks in the fission yeast fbp1 gene
Source: PLoS One. 2024 Jan 22;19(1):e0294191. doi: 10.1371/journal.pone.0294191 (PMC10802949; doi:10.1371/journal.pone.0294191)
Supplement: S1 Table — (PDF) [file pone.0294191.s001.pdf]

Supplementary Table S1

Strain Number	Genotype	Reference
SPH731	h^- *ade6-M26 ura4-D18 pat1-114 rec12-flag<<*Kan^R	This study
SPH815	h^- *ade6-M26 ura4-D18 pat1-114 rad50S*<br>*fbp1-mlonRNA-c initiation element replacement*	This study
SPH816	h^- *ade6-M26 ura4-D18 pat1-114 rad50S*<br>*fbp1-TATA-box mutation*	This study
SPH832	h^- *ade6-M26 ura4-D18 pat1-114 rad50S*<br>*fbp1-UAS1 mutation*	This study
SPH850	h^- *ade6-M26 ura4-D18 pat1-114 rad50S*<br>*fbp1-UAS2 mutation*	This study
SPH851	h^- *ade6-M26 ura4-D18 pat1-114 rad50S*	This study
SPH887	h^- *ade6-M26 ura4-D18 pat1-114 rad50S pcr1::ura4*	This study
