# Supplementary figures and images for: Metabolic stress-induced long ncRNA transcription governs the formation of meiotic DNA breaks in the fission yeast fbp1 gene
Source: PLoS One. 2024 Jan 22;19(1):e0294191. doi: 10.1371/journal.pone.0294191 (PMC10802949; doi:10.1371/journal.pone.0294191)

# Supplementary Fig. S1

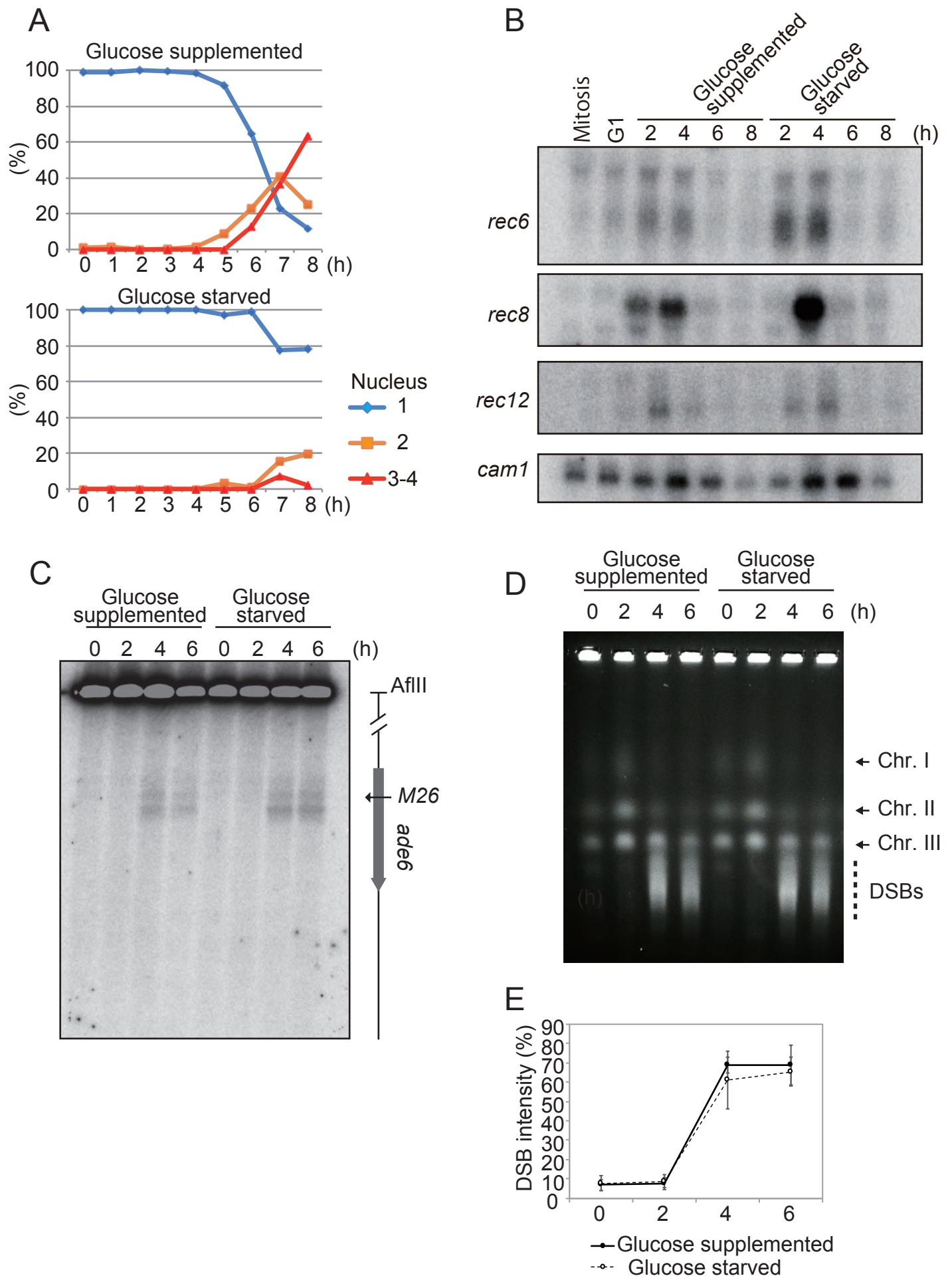

Supplement: S1 Fig — (A) Meiotic progressions of h- pat1-114 cells (SPH731) in glucose-supplemented and glucose-starved conditions were monitored by the observation of nuclear divisions. (B) Representative images of the Northern blot showing the expression of meiotic genes (rec6, rec8, and rec12) after the onset of meiosis with or without glucose. The cam1 transcript was used as an internal control. (C) Haploid pat1-114 rad50S cells (SPH851) were cultured to induce meiosis with or without glucose as in Fig 1B. Meiotic DSBs around the ade6-M26 locus were detected by Southern blotting. The arrow indicates the position of M26 mutation sites where the Atf1-Pcr1 transcription factor binds and induces DSBs. (D) Whole genome DNA including each intact chromosomal DNA and DSBs were separated with pulsed-field gel electrophoresis as in Fig 5. Dotted line indicates DSB signal. (E) Intensities of DSB signals in the indicated cells were quantified using Image J. (PDF) [file pone.0294191.s002.pdf]
