# Supplementary material for: Metabolic stress-induced long ncRNA transcription governs the formation of meiotic DNA breaks in the fission yeast fbp1 gene
Source: PLoS One. 2024 Jan 22;19(1):e0294191. doi: 10.1371/journal.pone.0294191 (PMC10802949; doi:10.1371/journal.pone.0294191)

Supplementary Fig. S2_1

Uncropped blot used in Fig 1B

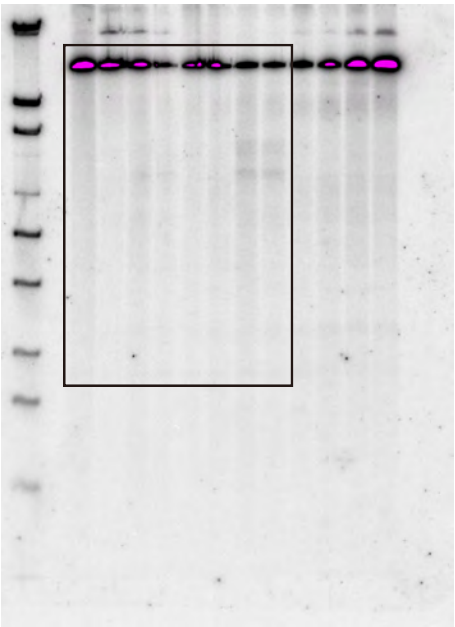

Uncropped blot used in Fig 1D

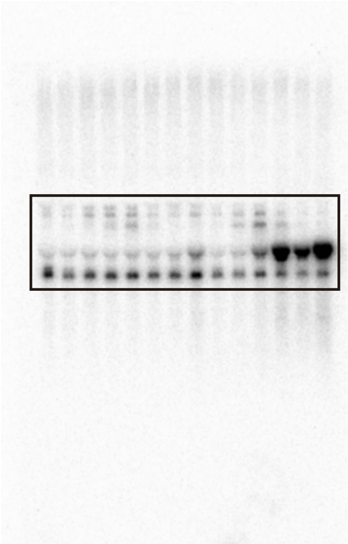

Uncropped blot used in Fig 1E

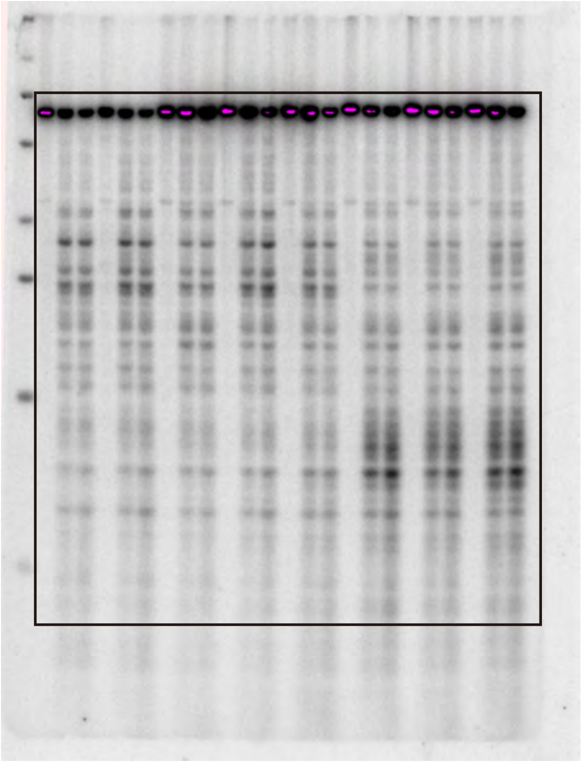

Uncropped blot used in Fig 2A

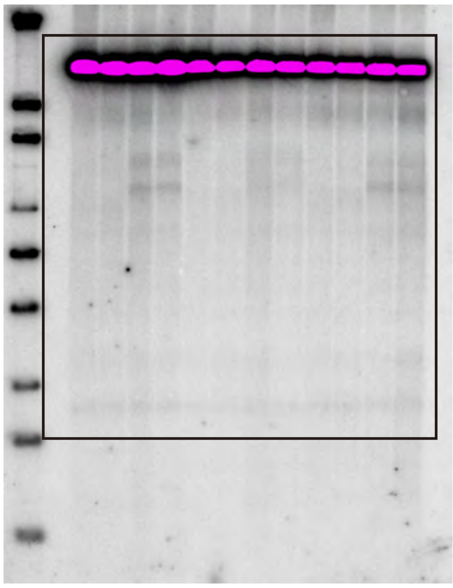

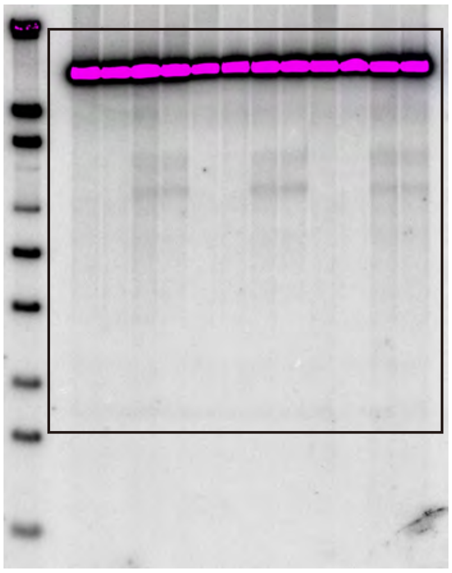

Supplementary Fig. S2_2

Uncropped blot used in Fig 3A

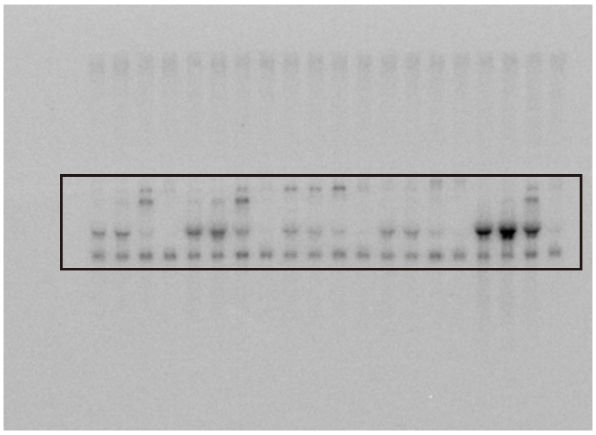

Uncropped blot used in Fig 3B

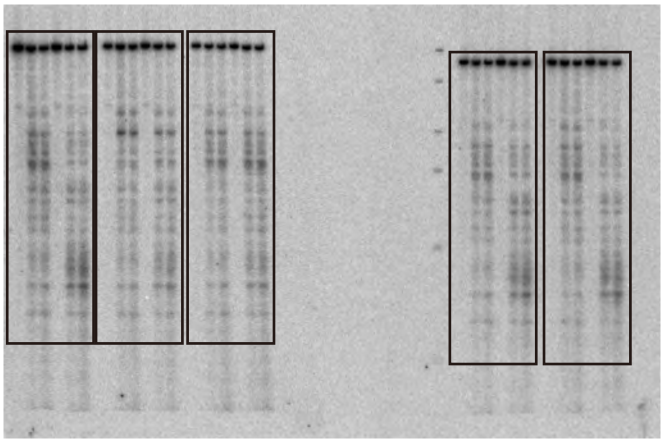

Uncropped blot used in Fig 5A

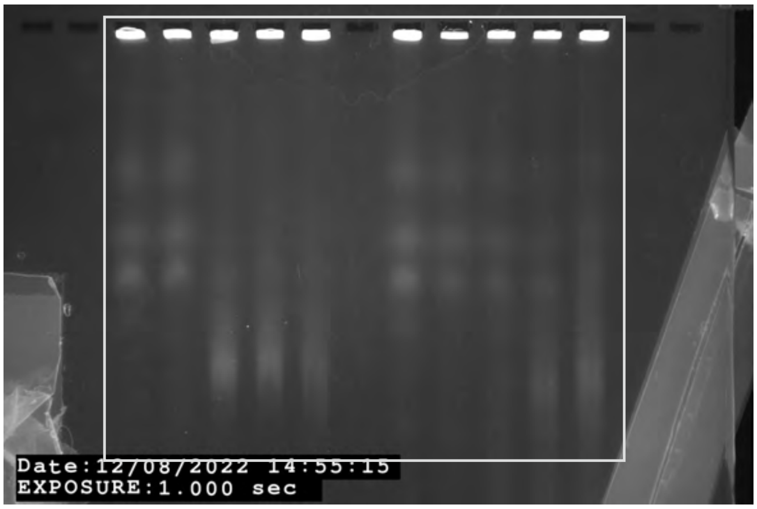

Uncropped blot used in Fig 5C

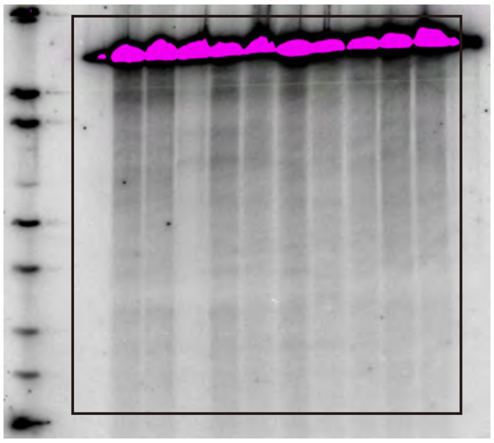

Uncropped blot used in Fig S1D

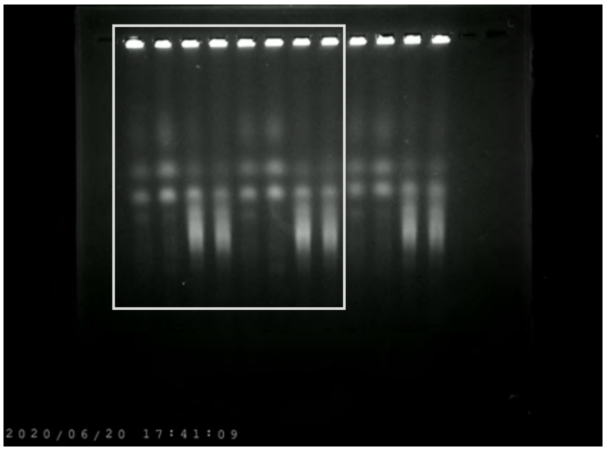

Uncropped blot used in Fig S1C

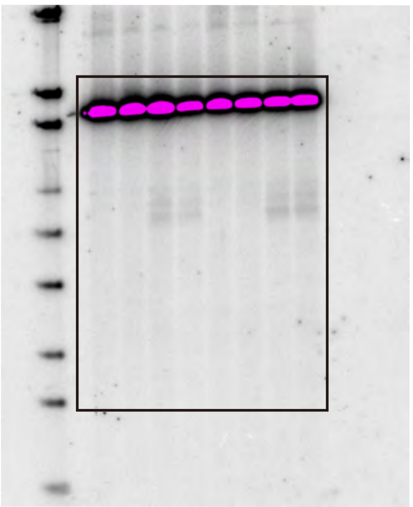

Uncropped blot used in Fig S1B

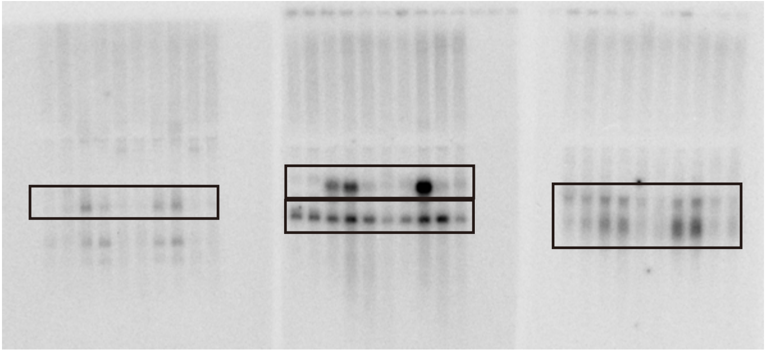

Supplement: S2 Fig — Full size images of raw blot data were shown in S1 Raw images. (PDF) [file pone.0294191.s003.pdf]
