# Supplementary material for: Metabolic stress-induced long ncRNA transcription governs the formation of meiotic DNA breaks in the fission yeast fbp1 gene
Source: PLoS One. 2024 Jan 22;19(1):e0294191. doi: 10.1371/journal.pone.0294191 (PMC10802949; doi:10.1371/journal.pone.0294191)
Supplement: S1 Raw images — (PDF) [file pone.0294191.s004.pdf]

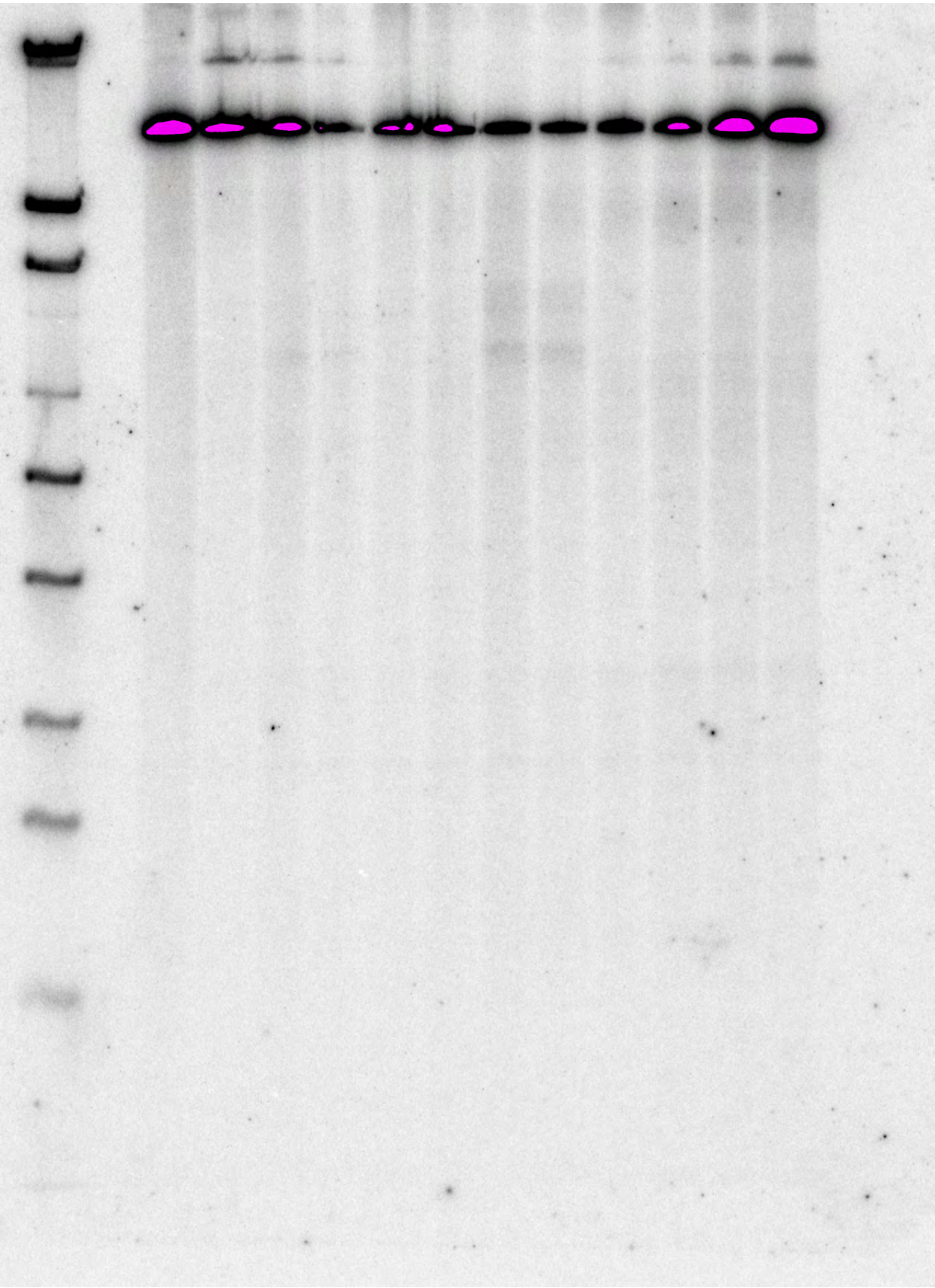

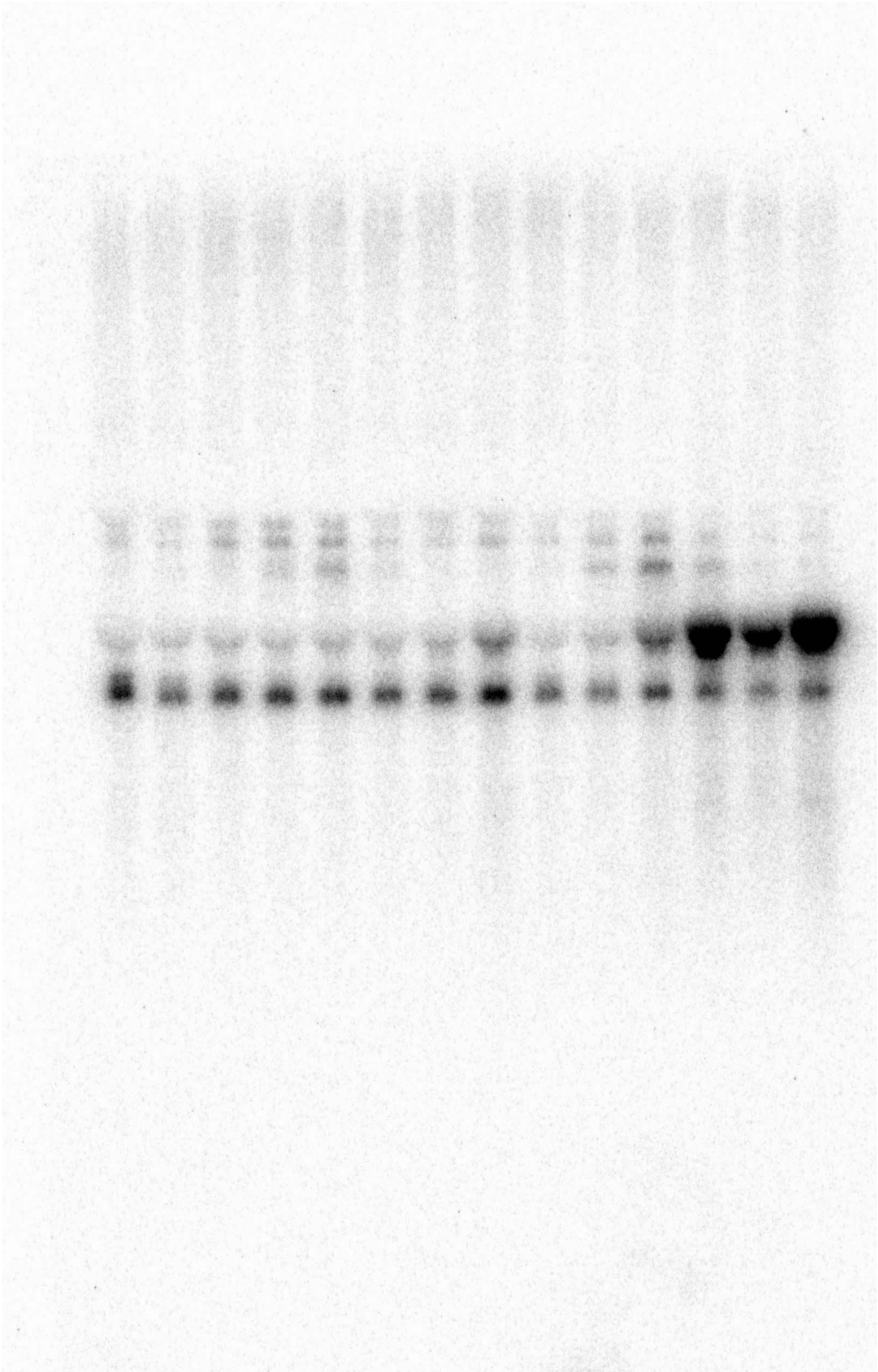

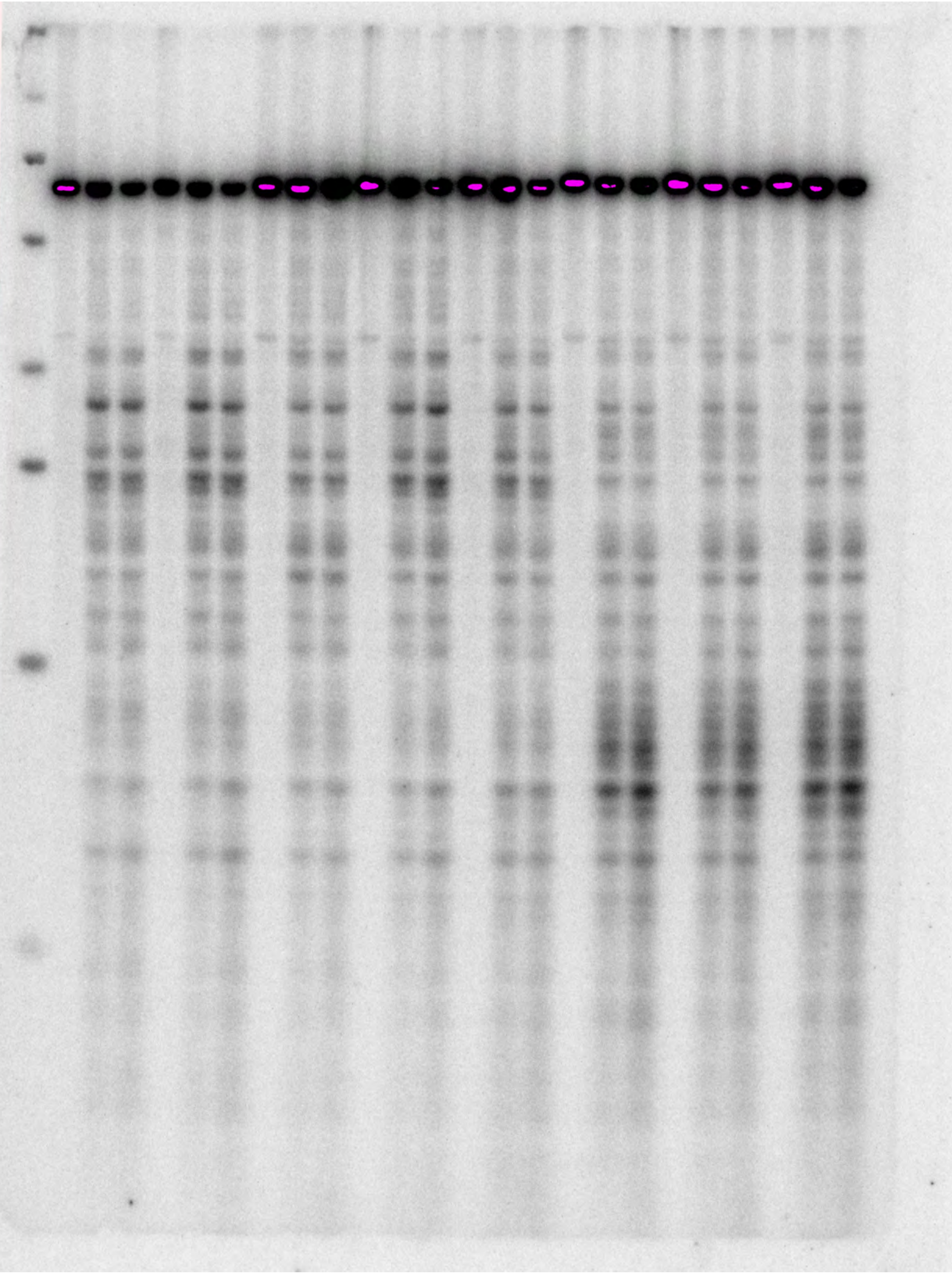

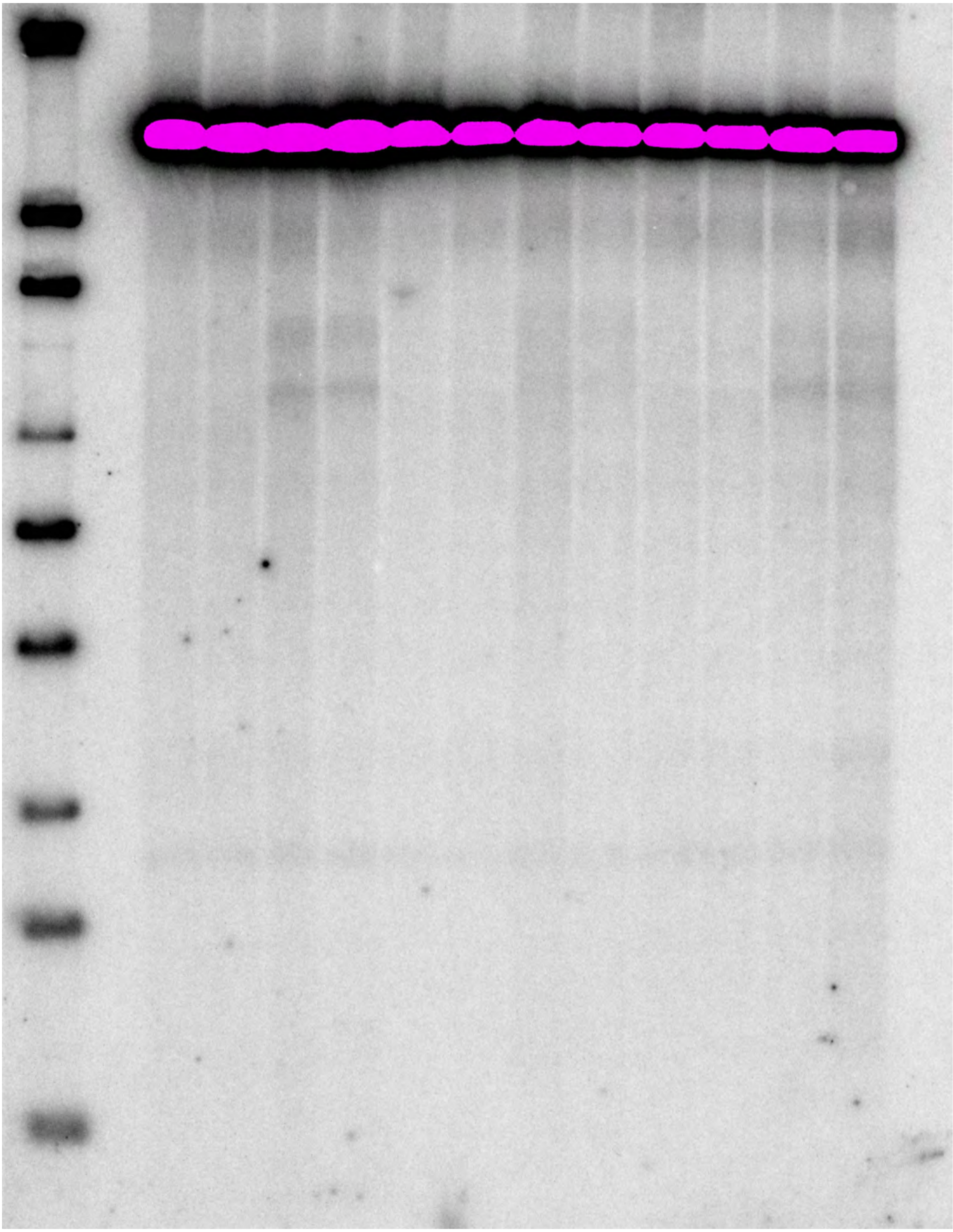

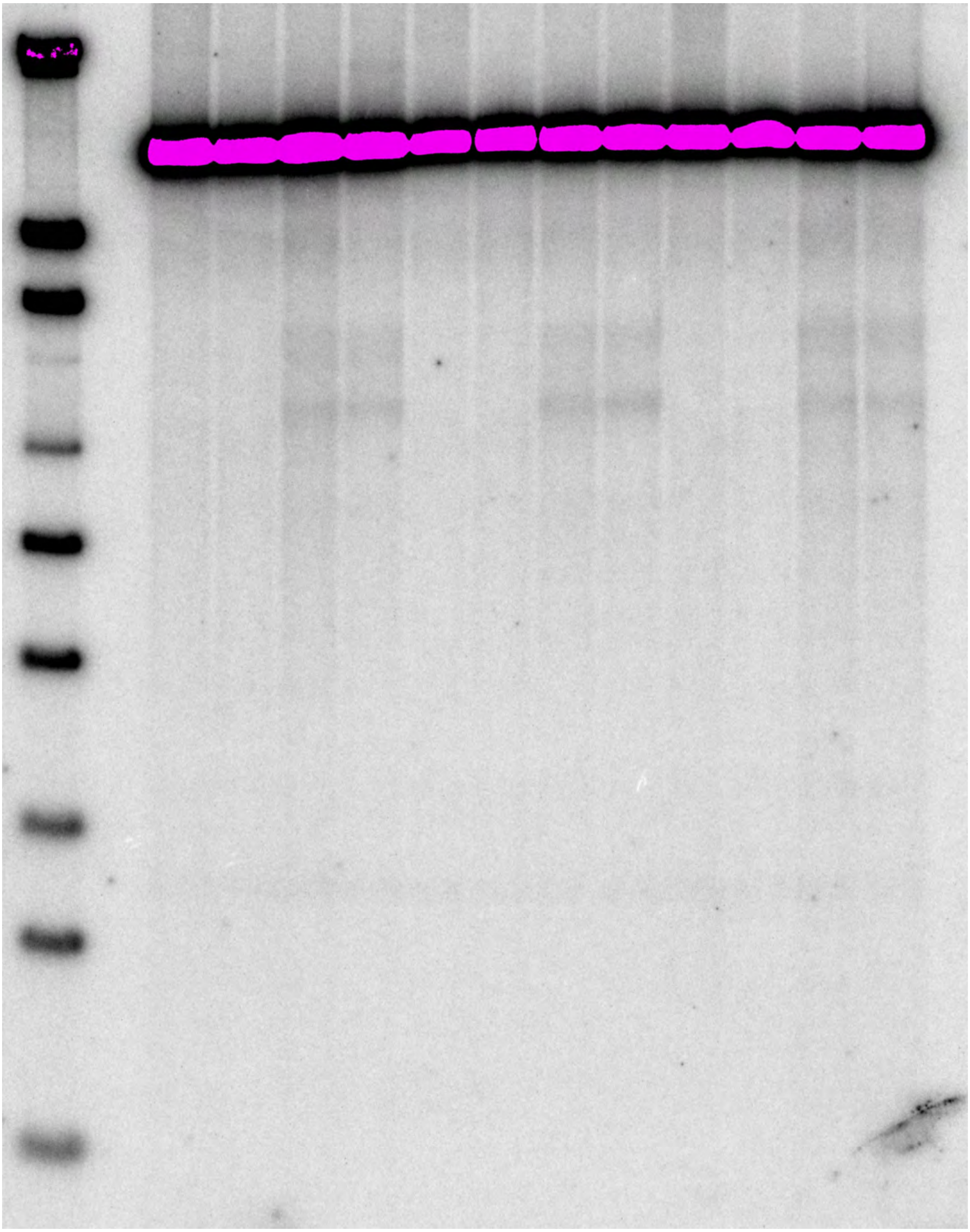

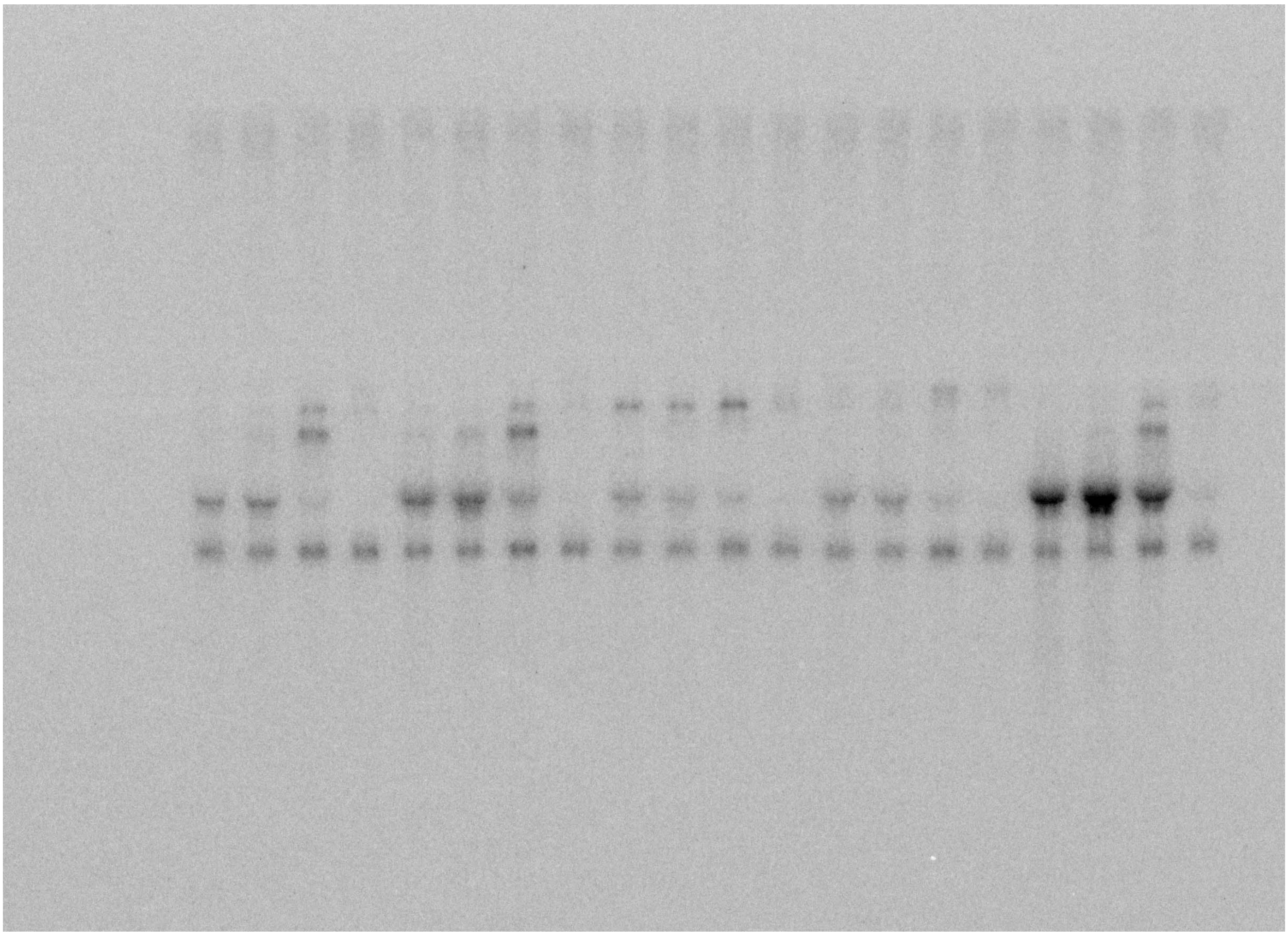

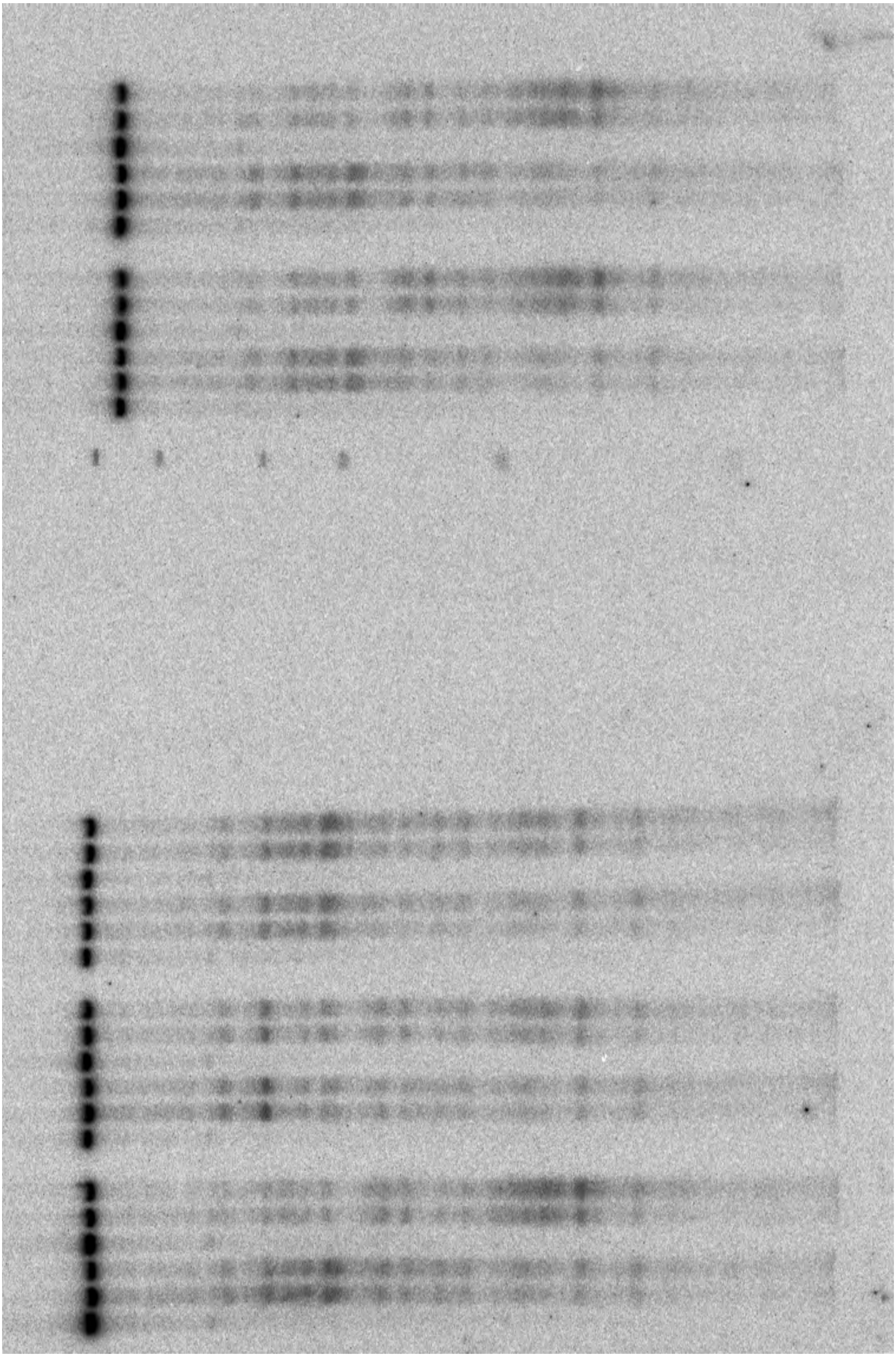

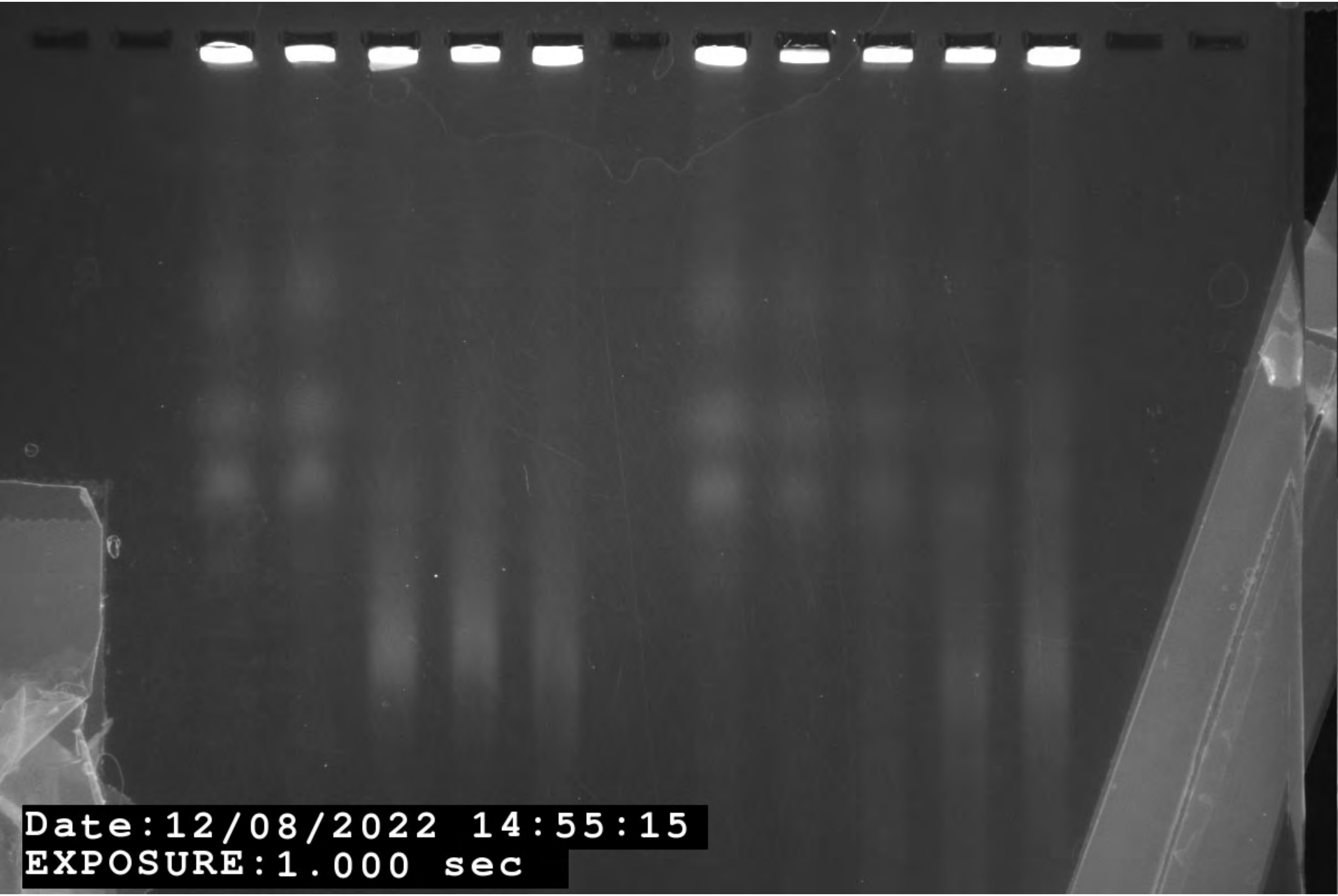
Date:12/08/2022 14:55:15
EXPOSURE:1.000 sec

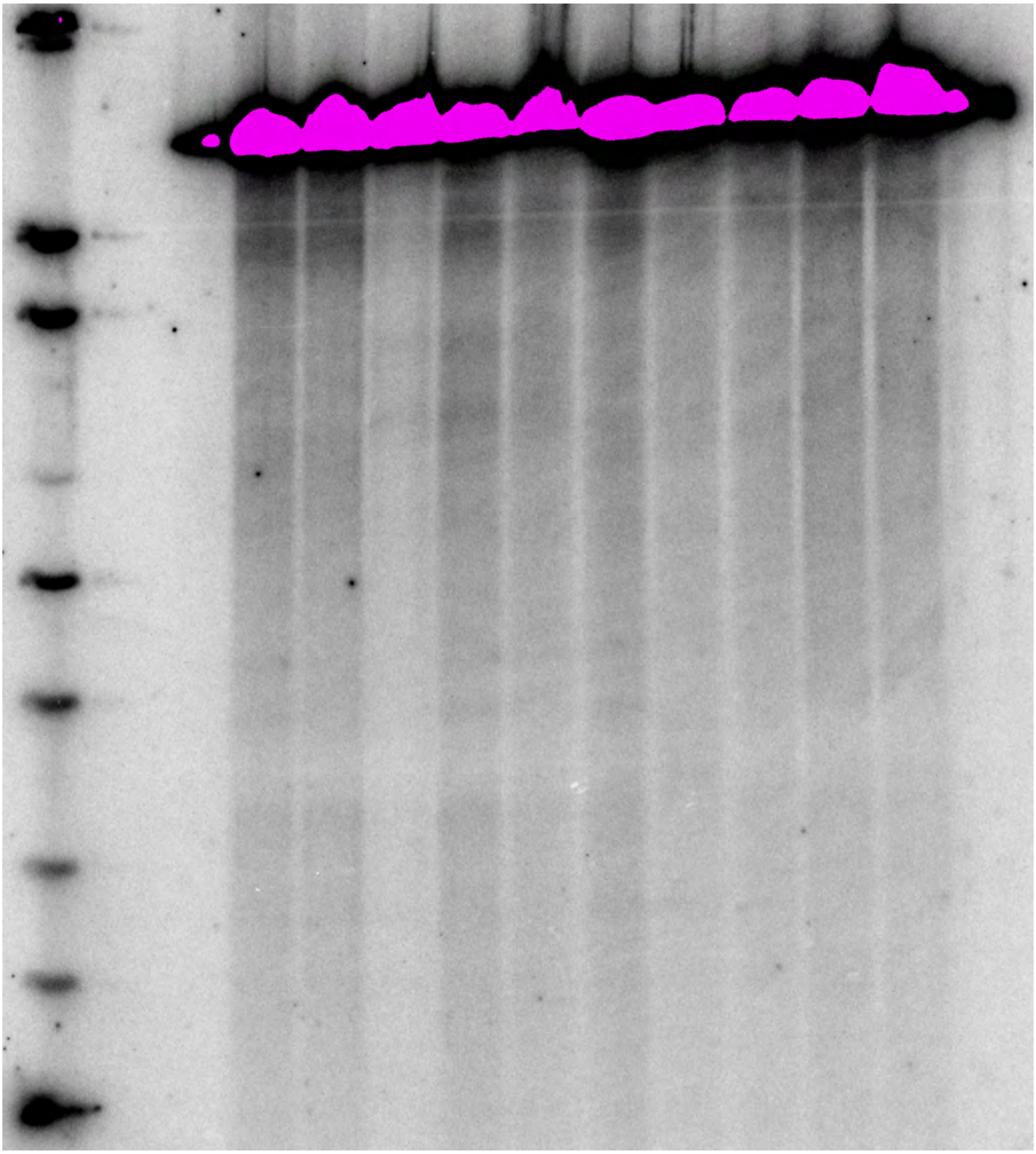

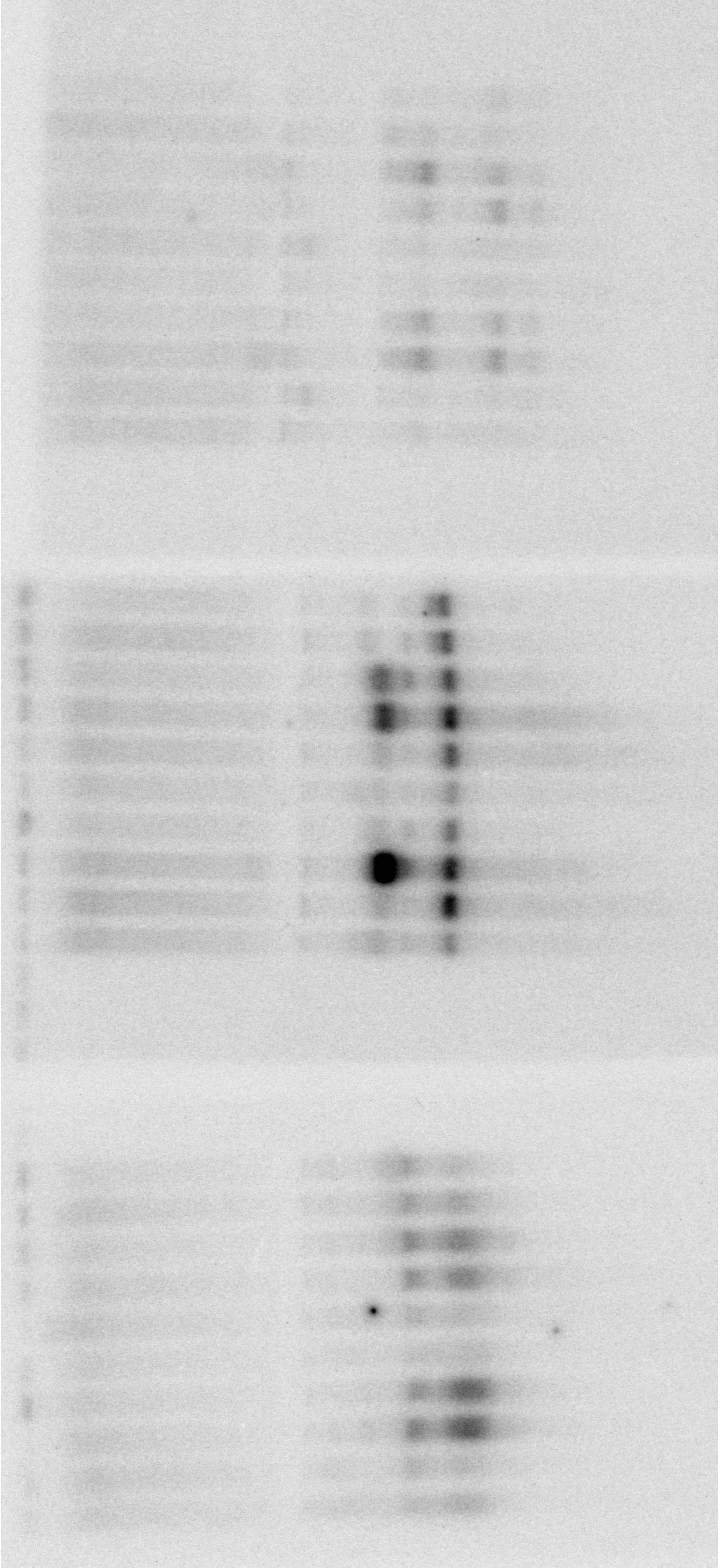

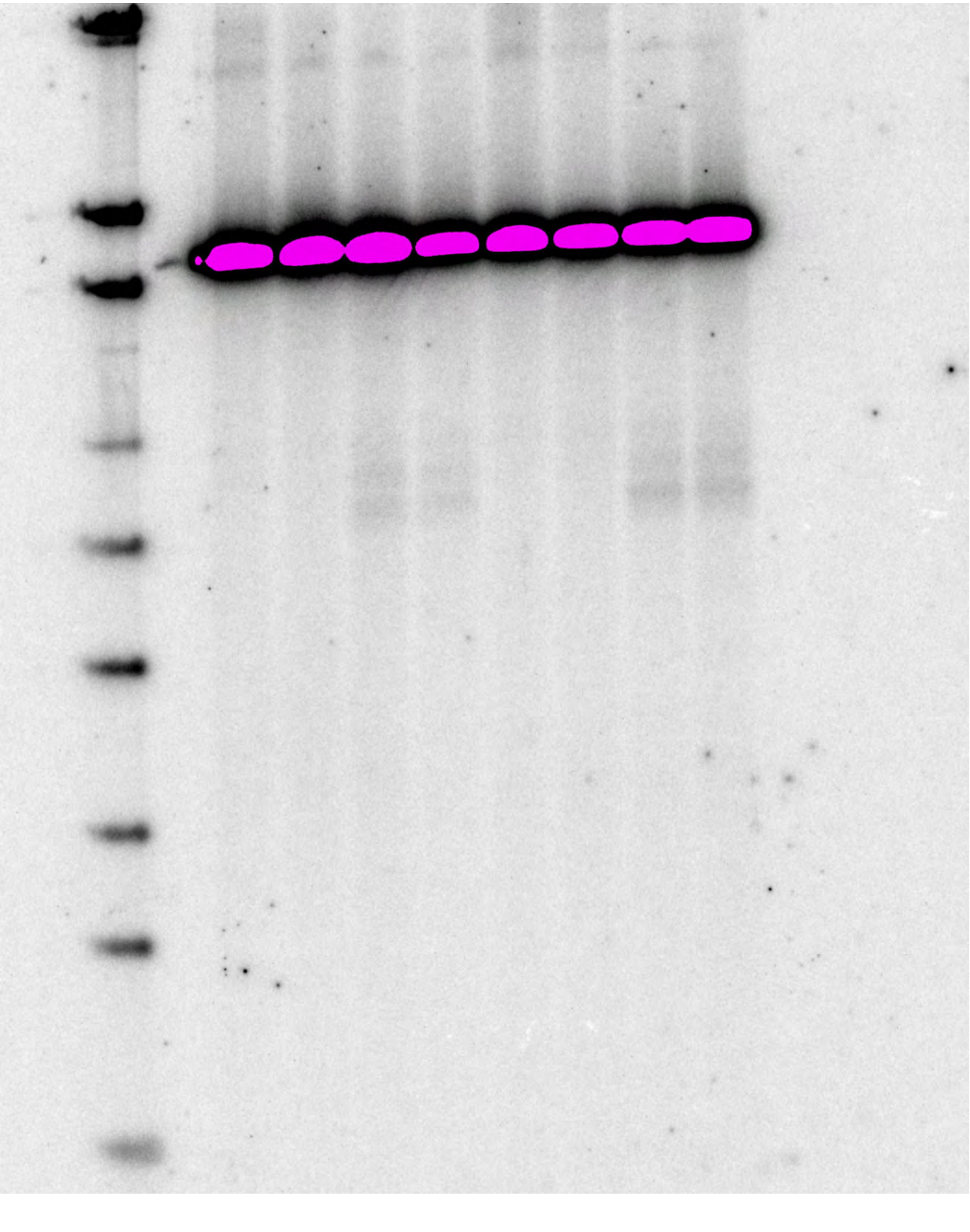

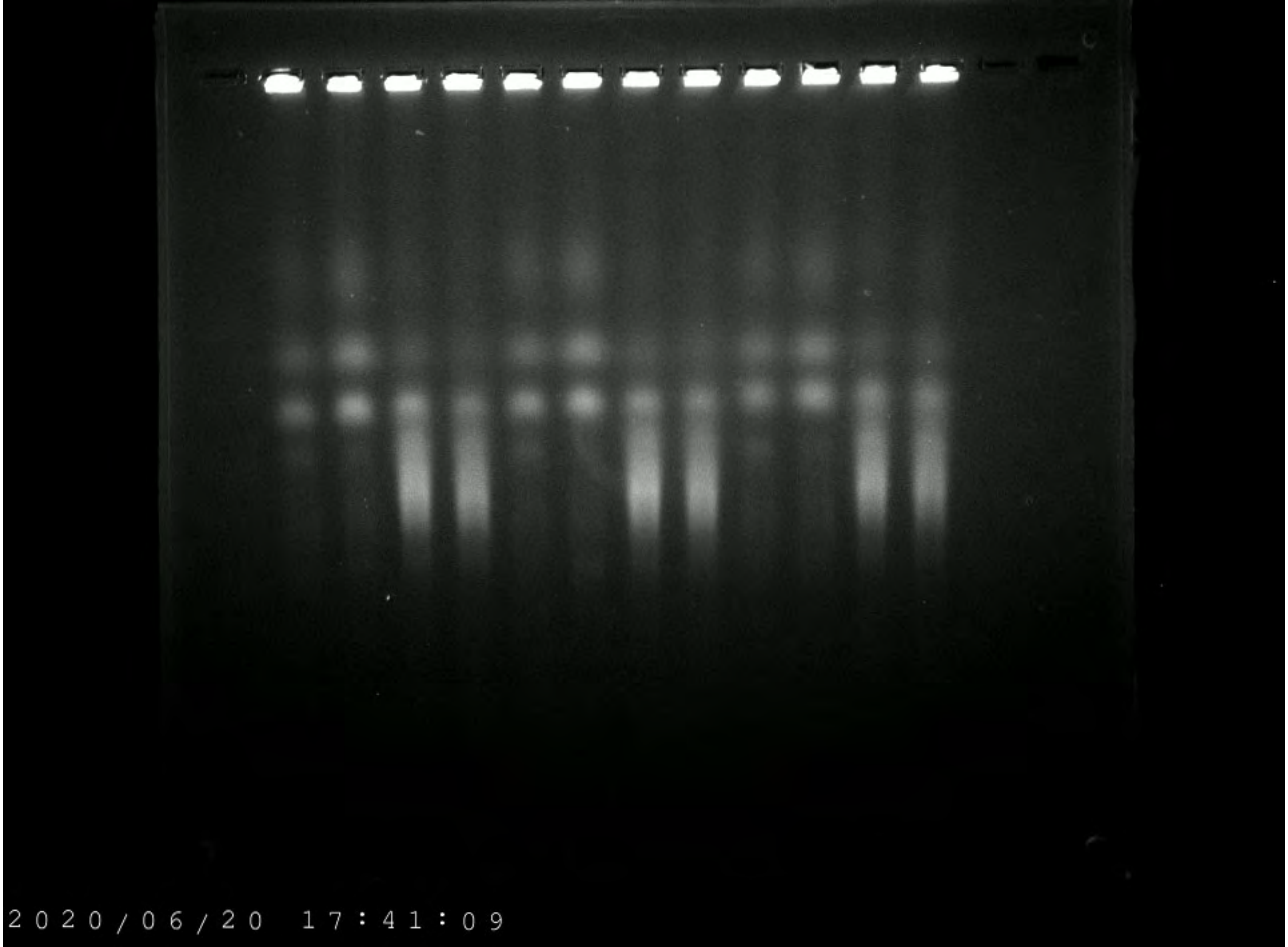
2020/06/20 17:41:09
